# New analysis for consistency among markers in the study of genetic diversity: development and application to the description of bacterial diversity

**DOI:** 10.1186/1471-2148-7-156

**Published:** 2007-09-03

**Authors:** Sandrine Pavoine, Xavier Bailly

**Affiliations:** 1 Unité de Conservation des espèces, restauration et suivi des populations (UMR MNHN-UPMC-CNRS 5173), Muséum National d'Histoire Naturelle, 55 rue Buffon, 75005 Paris, France; 2 Department of Biology, University of York, Post Office Box 373, York, YO10 5YW, UK

## Abstract

**Background:**

The development of post-genomic methods has dramatically increased the amount of qualitative and quantitative data available to understand how ecological complexity is shaped. Yet, new statistical tools are needed to use these data efficiently. In support of sequence analysis, diversity indices were developed to take into account both the relative frequencies of alleles and their genetic divergence. Furthermore, a method for describing inter-population nucleotide diversity has recently been proposed and named the double principal coordinate analysis (DPCoA), but this procedure can only be used with one locus. In order to tackle the problem of measuring and describing nucleotide diversity with more than one locus, we developed three versions of multiple DPCoA by using three ordination methods: multiple co-inertia analysis, STATIS, and multiple factorial analysis.

**Results:**

This combination of methods allows i) testing and describing differences in patterns of inter-population diversity among loci, and ii) defining the best compromise among loci. These methods are illustrated by the analysis of both simulated data sets, which include ten loci evolving under a stepping stone model and a locus evolving under an alternative population structure, and a real data set focusing on the genetic structure of two nitrogen fixing bacteria, which is influenced by geographical isolation and host specialization. All programs needed to perform multiple DPCoA are freely available.

**Conclusion:**

Multiple DPCoA allows the evaluation of the impact of various loci in the measurement and description of diversity. This method is general enough to handle a large variety of data sets. It complements existing methods such as the analysis of molecular variance or other analyses based on linkage disequilibrium measures, and is very useful to study the impact of various loci on the measurement of diversity.

## Background

The exponential increase in sequencing abilities is modifying the way genetic diversity is assessed. For instance, multilocus sequencing (MLS) now allows the estimation of genetic relatedness among microorganisms for both housekeeping genes and accessory genes such as virulence or symbiotic determinants [1]. Thus, several publications reported complex MLS schemes studying more than ten genes located in different genomic regions and involved in various metabolic pathways. These studies have indicated the influence of various parameters, such as recombination rate [2] or epidemiological traits [3], on the diversification of bacterial populations. Furthermore, recent progress in sequencing technologies suggests that still more and more sequence data will be available to study questions related to community ecology in the near future [4]. New statistical methodologies should therefore be developed to deal with the complexity of data sets that will be produced. One of the main problems raised by the increase in sequence information is the assessment of congruence among population structures depicted by different molecular markers [5]. In bacterial lineages, especially for those in which sex is common, the diversity of each locus could be shaped by the gain/loss of genes, gene flow boundaries and specific selective pressures [6]. The problems which can arise from the overall analysis of a MLS data set in which loci do not share congruent evolutionary constraints include, among others, misleading inferences of genetic relatedness and phylogenetic relationships [7] or overestimation of linkage disequilibrium [8].

Bacterial isolates which are characterized by MLS usually belong to several genetic groups (*i.e*. species or populations) which can be defined according to the sampling strategy or according to more refined methodologies [9]. For each locus of a MLS data set, the different sequence types recovered are called alleles. In this context, the properties of the data set can be summarized by two sets of matrices. The first set includes *G *matrices {**F**_1_,..., **F**_*g*_,..., **F**_*G*_}, in which *G *is the number of loci. Each of these matrices contains the frequencies of the different alleles recovered at a given locus among the populations under study. The dimensions of these matrices are thus (*ρ*_1_, *r*), ..., (*ρ*_*g*_, *r*), ..., (*ρ*_*G*_, *r*), in which *ρ*_*g *_is the number of alleles observed at locus *g *and *r *is the number of populations delineated. The second set also includes *G *matrices called {**D**_1_,..., **D**_*g*_..., **D**_*G*_}, which contain the pairwise genetic distances between the alleles observed at locus *g*. Usually, the information contained within these two sets of matrices are analyzed independently using respective population genetic statistics (*i.e*. diversity indices and differentiation measures) and phylogenetic methods. Yet, while it is possible to perform analyses over all loci in either a population genetic or a phylogenetic framework, few methodologies are available to assess the congruence of the information obtained from different loci. In particular, a comparison of the patterns revealed by differentiation measures among the populations sampled, *i.e*. population structure, is a problematic issue.

Multivariate analysis is an interesting methodological way to approach this problem. For instance, Moazami-Goudarzi and Laloë [5] have proposed a two-step procedure to test the dissimilarity in population structures revealed by different microsatellite loci. Although this analysis can be used to test the similarity of population differentiations inferred from a set of markers, it can be noted that: i) it can not be used to describe population structures, and ii) genetic divergence among alleles are not taken into account, while these can be quite informative. Consequently, further improvements should be considered since alternative statistical approaches are available [10]. In this context, the aim of this survey is to propose a new procedure called multiple double principal coordinate analyses (mDPCoA). The mDPCoA aims at comparing inter-population structures provided by the different markers of a MLS scheme. Firstly, a pattern of population differences is obtained for each MLS marker using a double principal coordinate analysis (DPCoA) which is a recently developed ordination method which takes into account both the frequency of alleles and their genetic divergence [11] (see Eckburg *et al*. [12] and Bik *et al*. [13] for applications of this method to the analysis of bacterial diversity). Secondly, population patterns are compared using three different methods: the Multiple Co-inertia Analysis [14], STATIS [15], and the Multiple Factorial Analysis [16]. Finally, a permutation procedure can be used to test the pairwise correlation among MLS markers. These analysis pipelines have been used on either simulated or published MLS data sets to check the accuracy and the relevance of the procedures. The results obtained illustrate the ability of this methodology to make inferences on various features of populations under study.

## Results

### Algorithms of multiple Double Principal Coordinate Analysis

Computations were performed using new functions and functions implemented in the ade4 [17] and ape [18] packages written in the R software [19] [see Additional file 1]. A manual describing the use of the different functions is supplied [see Additional file 2].

Let {**F**_1_,..., **F**_*g*_,..., **F**_*G *_be the set of matrices of type alleles × populations, containing the frequencies of alleles in the populations for the *G *loci, {**D**_1_,..., **D**_*g*_,..., **D**_*G*_} be the set of matrices containing the distances among alleles, **B**_*r *_be the diagonal matrix containing the population weights (the weight of a population is the proportion of individuals drawn from this population), and Bρg
 MathType@MTEF@5@5@+=feaafiart1ev1aaatCvAUfKttLearuWrP9MDH5MBPbIqV92AaeXatLxBI9gBaebbnrfifHhDYfgasaacH8akY=wiFfYdH8Gipec8Eeeu0xXdbba9frFj0=OqFfea0dXdd9vqai=hGuQ8kuc9pgc9s8qqaq=dirpe0xb9q8qiLsFr0=vr0=vr0dc8meaabaqaciaacaGaaeqabaqabeGadaaakeaacqWHcbGqdaWgaaWcbaacciGae8xWdi3aaSbaaWqaaiabdEgaNbqabaaaleqaaaaa@313F@ be the diagonal matrix containing the allele weights for the *g*^th ^locus (the weight of an allele is its frequency over all the populations studied). The matrices of distances must be Euclidean [20], which is obtained with, for example, either Lingoes [21] or Cailliez [22] correction.

For a single locus *g*, the analysis of the among-population diversity corresponds to a DPCoA, which results in three main steps:

1. Defining a Euclidean space composed by principal axes of the distances among the alleles. The coordinates of the alleles in this space are in **R**_*g *_such that: −QgtDgQg=RgRgt
 MathType@MTEF@5@5@+=feaafiart1ev1aaatCvAUfKttLearuWrP9MDH5MBPbIqV92AaeXatLxBI9gBaebbnrfifHhDYfgasaacH8akY=wiFfYdH8Gipec8Eeeu0xXdbba9frFj0=OqFfea0dXdd9vqai=hGuQ8kuc9pgc9s8qqaq=dirpe0xb9q8qiLsFr0=vr0=vr0dc8meaabaqaciaacaGaaeqabaqabeGadaaakeaacqGHsislcqWHrbqudaqhaaWcbaGaem4zaCgabaGaemiDaqhaaOGaeCiraq0aaSbaaSqaaiabdEgaNbqabaGccqWHrbqudaWgaaWcbaGaem4zaCgabeaakiabg2da9iabhkfasnaaBaaaleaacqWGNbWzaeqaaOGaeCOuai1aa0baaSqaaiabdEgaNbqaaiabdsha0baaaaa@3F0F@, where Qg=Iρg−Bρg1ρg1ρgt
 MathType@MTEF@5@5@+=feaafiart1ev1aaatCvAUfKttLearuWrP9MDH5MBPbIqV92AaeXatLxBI9gBaebbnrfifHhDYfgasaacH8akY=wiFfYdH8Gipec8Eeeu0xXdbba9frFj0=OqFfea0dXdd9vqai=hGuQ8kuc9pgc9s8qqaq=dirpe0xb9q8qiLsFr0=vr0=vr0dc8meaabaqaciaacaGaaeqabaqabeGadaaakeaacqWHrbqudaWgaaWcbaGaem4zaCgabeaakiabg2da9iabhMeajnaaBaaaleaaiiGacqWFbpGCdaWgaaadbaGaem4zaCgabeaaaSqabaGccqGHsislcqWHcbGqdaWgaaWcbaGae8xWdi3aaSbaaWqaaiabdEgaNbqabaaaleqaaOGaeCymaeZaaSbaaSqaaiab=f8aYnaaBaaameaacqWGNbWzaeqaaaWcbeaakiabhgdaXmaaDaaaleaacqWFbpGCdaWgaaadbaGaem4zaCgabeaaaSqaaiabdsha0baaaaa@44DD@ is a projector which proceeds to weighted centering, with Iρg
 MathType@MTEF@5@5@+=feaafiart1ev1aaatCvAUfKttLearuWrP9MDH5MBPbIqV92AaeXatLxBI9gBaebbnrfifHhDYfgasaacH8akY=wiFfYdH8Gipec8Eeeu0xXdbba9frFj0=OqFfea0dXdd9vqai=hGuQ8kuc9pgc9s8qqaq=dirpe0xb9q8qiLsFr0=vr0=vr0dc8meaabaqaciaacaGaaeqabaqabeGadaaakeaacqWHjbqsdaWgaaWcbaacciGae8xWdi3aaSbaaWqaaiabdEgaNbqabaaaleqaaaaa@314D@ the *ρ*_*g *_× *ρ*_*g *_matrix of identity and 1ρg
 MathType@MTEF@5@5@+=feaafiart1ev1aaatCvAUfKttLearuWrP9MDH5MBPbIqV92AaeXatLxBI9gBaebbnrfifHhDYfgasaacH8akY=wiFfYdH8Gipec8Eeeu0xXdbba9frFj0=OqFfea0dXdd9vqai=hGuQ8kuc9pgc9s8qqaq=dirpe0xb9q8qiLsFr0=vr0=vr0dc8meaabaqaciaacaGaaeqabaqabeGadaaakeaacqWHXaqmdaWgaaWcbaacciGae8xWdi3aaSbaaWqaaiabdEgaNbqabaaaleqaaaaa@311D@ a *ρ*_*g *_× 1 vector of units. That is to say, QgtDgQg
 MathType@MTEF@5@5@+=feaafiart1ev1aaatCvAUfKttLearuWrP9MDH5MBPbIqV92AaeXatLxBI9gBaebbnrfifHhDYfgasaacH8akY=wiFfYdH8Gipec8Eeeu0xXdbba9frFj0=OqFfea0dXdd9vqai=hGuQ8kuc9pgc9s8qqaq=dirpe0xb9q8qiLsFr0=vr0=vr0dc8meaabaqaciaacaGaaeqabaqabeGadaaakeaacqWHrbqudaqhaaWcbaGaem4zaCgabaGaemiDaqhaaOGaeCiraq0aaSbaaSqaaiabdEgaNbqabaGccqWHrbqudaWgaaWcbaGaem4zaCgabeaaaaa@362E@ is the matrix centered by rows and columns;

2. Positioning, in this space, the populations at the centroid of the alleles they possess. The coordinates of the populations, in this space, are in **C**_*g *_such that: Cg=Br−1FgtRg
 MathType@MTEF@5@5@+=feaafiart1ev1aaatCvAUfKttLearuWrP9MDH5MBPbIqV92AaeXatLxBI9gBaebbnrfifHhDYfgasaacH8akY=wiFfYdH8Gipec8Eeeu0xXdbba9frFj0=OqFfea0dXdd9vqai=hGuQ8kuc9pgc9s8qqaq=dirpe0xb9q8qiLsFr0=vr0=vr0dc8meaabaqaciaacaGaaeqabaqabeGadaaakeaacqWHdbWqdaWgaaWcbaGaem4zaCgabeaakiabg2da9iabhkeacnaaDaaaleaacqWGYbGCaeaacqGHsislcqaIXaqmaaGccqWHgbGrdaqhaaWcbaGaem4zaCgabaGaemiDaqhaaOGaeCOuai1aaSbaaSqaaiabdEgaNbqabaaaaa@3BB0@;

3. Proceeding to the singular value decomposition of the triplet (**C**_*g*_, Iμg
 MathType@MTEF@5@5@+=feaafiart1ev1aaatCvAUfKttLearuWrP9MDH5MBPbIqV92AaeXatLxBI9gBaebbnrfifHhDYfgasaacH8akY=wiFfYdH8Gipec8Eeeu0xXdbba9frFj0=OqFfea0dXdd9vqai=hGuQ8kuc9pgc9s8qqaq=dirpe0xb9q8qiLsFr0=vr0=vr0dc8meaabaqaciaacaGaaeqabaqabeGadaaakeaacqWHjbqsdaWgaaWcbaacciGae8hVd02aaSbaaWqaaiabdEgaNbqabaaaleqaaaaa@3143@, **B**_*r*_), where *μ*_*g *_is the number of principal axes for the alleles of the *g*^th ^locus. This third step leads to a set of positive eigenvalues, in a diagonal (*ν*_*g *_× *ν*_*g*_) matrix **Ψ**_*g*_, and to a base of orthonormal eigenvectors, in a (*r *× *ν*_*g*_) matrix **V**_*g*_, defining the new Euclidean space. The eigenvectors constitute the principal axes of the distances among populations. In this new space, which is the DPCoA space, the coordinates of the alleles are in **X**_*g *_= **R**_*g*_**V**_*g*_, and the coordinates of the populations in **Y**_*g *_= **C**_*g*_**V**_*g*_.

A consideration of the set of all the loci leads thus to *G *triplets (Y1,Iν1,Br),...,(Yg,Iνg,Br),...,(YG,IνG,Br)
 MathType@MTEF@5@5@+=feaafiart1ev1aaatCvAUfKttLearuWrP9MDH5MBPbIqV92AaeXatLxBI9gBaebbnrfifHhDYfgasaacH8akY=wiFfYdH8Gipec8Eeeu0xXdbba9frFj0=OqFfea0dXdd9vqai=hGuQ8kuc9pgc9s8qqaq=dirpe0xb9q8qiLsFr0=vr0=vr0dc8meaabaqaciaacaGaaeqabaqabeGadaaakeaadaqadaqaaiabhMfaznaaBaaaleaacqaIXaqmaeqaaOGaeiilaWIaeCysaK0aaSbaaSqaaGGaciab=17aUnaaBaaameaacqaIXaqmaeqaaaWcbeaakiabcYcaSiabhkeacnaaBaaaleaacqWGYbGCaeqaaaGccaGLOaGaayzkaaGaeiilaWIaeiOla4IaeiOla4IaeiOla4IaeiilaWYaaeWaaeaacqWHzbqwdaWgaaWcbaGaem4zaCgabeaakiabcYcaSiabhMeajnaaBaaaleaacqWF9oGBdaWgaaadbaGaem4zaCgabeaaaSqabaGccqGGSaalcqWHcbGqdaWgaaWcbaGaemOCaihabeaaaOGaayjkaiaawMcaaiabcYcaSiabc6caUiabc6caUiabc6caUiabcYcaSmaabmaabaGaeCywaK1aaSbaaSqaaiabdEeahbqabaGccqGGSaalcqWHjbqsdaWgaaWcbaGae8xVd42aaSbaaWqaaiabdEeahbqabaaaleqaaOGaeiilaWIaeCOqai0aaSbaaSqaaiabdkhaYbqabaaakiaawIcacaGLPaaaaaa@5C62@

Our objective being to evaluate the consistency among the patterns of inter-population diversity provided by each locus, considering evolutionary distances among alleles, we had to find a Euclidean space allowing the direct comparison among the individual DPCoA analyses. We evaluated three alternative solutions taken from the *K*-table multivariate analysis: the multiple co-inertia analysis (MCoA) [14], STATIS [15] and the multiple factorial analysis (MFA) [16].

### DPCoA and Multiple Co-inertia analysis

The Multiple Co-inertia Analysis applied to the triplets (Y1,Iν1,Br),...,(Yg,Iνg,Br),...,(YG,IνG,Br)
 MathType@MTEF@5@5@+=feaafiart1ev1aaatCvAUfKttLearuWrP9MDH5MBPbIqV92AaeXatLxBI9gBaebbnrfifHhDYfgasaacH8akY=wiFfYdH8Gipec8Eeeu0xXdbba9frFj0=OqFfea0dXdd9vqai=hGuQ8kuc9pgc9s8qqaq=dirpe0xb9q8qiLsFr0=vr0=vr0dc8meaabaqaciaacaGaaeqabaqabeGadaaakeaadaqadaqaaiabhMfaznaaBaaaleaacqaIXaqmaeqaaOGaeiilaWIaeCysaK0aaSbaaSqaaGGaciab=17aUnaaBaaameaacqaIXaqmaeqaaaWcbeaakiabcYcaSiabhkeacnaaBaaaleaacqWGYbGCaeqaaaGccaGLOaGaayzkaaGaeiilaWIaeiOla4IaeiOla4IaeiOla4IaeiilaWYaaeWaaeaacqWHzbqwdaWgaaWcbaGaem4zaCgabeaakiabcYcaSiabhMeajnaaBaaaleaacqWF9oGBdaWgaaadbaGaem4zaCgabeaaaSqabaGccqGGSaalcqWHcbGqdaWgaaWcbaGaemOCaihabeaaaOGaayjkaiaawMcaaiabcYcaSiabc6caUiabc6caUiabc6caUiabcYcaSmaabmaabaGaeCywaK1aaSbaaSqaaiabdEeahbqabaGccqGGSaalcqWHjbqsdaWgaaWcbaGae8xVd42aaSbaaWqaaiabdEeahbqabaaaleqaaOGaeiilaWIaeCOqai0aaSbaaSqaaiabdkhaYbqabaaakiaawIcacaGLPaaaaaa@5C62@.

can be viewed as follows:

The main step is the definition of a set of axes ug[k]
 MathType@MTEF@5@5@+=feaafiart1ev1aaatCvAUfKttLearuWrP9MDH5MBPbIqV92AaeXatLxBI9gBaebbnrfifHhDYfgasaacH8akY=wiFfYdH8Gipec8Eeeu0xXdbba9frFj0=OqFfea0dXdd9vqai=hGuQ8kuc9pgc9s8qqaq=dirpe0xb9q8qiLsFr0=vr0=vr0dc8meaabaqaciaacaGaaeqabaqabeGadaaakeaacqWH1bqDdaqhaaWcbaGaem4zaCgabaWaamWaaeaacqWGRbWAaiaawUfacaGLDbaaaaaaaa@32F8@, for 1 ≤ *k *<*K*, and 1 ≤ *g *≤ *G*, normalized in each space ℝνg
 MathType@MTEF@5@5@+=feaafiart1ev1aaatCvAUfKttLearuWrP9MDH5MBPbIqV92AaeXatLxBI9gBaebbnrfifHhDYfgasaacH8akY=wiFfYdH8Gipec8Eeeu0xXdbba9frFj0=OqFfea0dXdd9vqai=hGuQ8kuc9pgc9s8qqaq=dirpe0xb9q8qiLsFr0=vr0=vr0dc8meaabaqaciaacaGaaeqabaqabeGadaaakeaacqWIDesOdaahaaWcbeqaaGGaciab=17aUnaaBaaameaacqWGNbWzaeqaaaaaaaa@318C@, which will serve to position the populations according to each individual locus, and *K *unique variables **v**^[*k*]^, for 1 ≤ *k *<*K*, **D**_*r*_-normalized in ℝ^*r*^, which may be used to synthesize the information provided by the *G *loci. This definition is done by maximizing

∑g=1Gπg〈Ygug|v〉Br2
 MathType@MTEF@5@5@+=feaafiart1ev1aaatCvAUfKttLearuWrP9MDH5MBPbIqV92AaeXatLxBI9gBaebbnrfifHhDYfgasaacH8akY=wiFfYdH8Gipec8Eeeu0xXdbba9frFj0=OqFfea0dXdd9vqai=hGuQ8kuc9pgc9s8qqaq=dirpe0xb9q8qiLsFr0=vr0=vr0dc8meaabaqaciaacaGaaeqabaqabeGadaaakeaadaaeWbqaaGGaciab=b8aWnaaBaaaleaacqWGNbWzaeqaaOWaaaWaaeaacqWHzbqwdaWgaaWcbaGaem4zaCgabeaakiabhwha1naaBaaaleaacqWGNbWzaeqaaOGaeiiFaWNaeCODayhacaGLPmIaayPkJaWaa0baaSqaaiabhkeacnaaBaaameaacqWGYbGCaeqaaaWcbaGaeGOmaidaaaqaaiabdEgaNjabg2da9iabigdaXaqaaiabdEeahbqdcqGHris5aaaa@4506@, given that

〈v[k]|v[l]〉Br=0
 MathType@MTEF@5@5@+=feaafiart1ev1aaatCvAUfKttLearuWrP9MDH5MBPbIqV92AaeXatLxBI9gBaebbnrfifHhDYfgasaacH8akY=wiFfYdH8Gipec8Eeeu0xXdbba9frFj0=OqFfea0dXdd9vqai=hGuQ8kuc9pgc9s8qqaq=dirpe0xb9q8qiLsFr0=vr0=vr0dc8meaabaqaciaacaGaaeqabaqabeGadaaakeaadaaadaqaaiabhAha2naaCaaaleqabaWaamWaaeaacqWGRbWAaiaawUfacaGLDbaaaaGccqGG8baFcqWH2bGDdaahaaWcbeqaamaadmaabaGaemiBaWgacaGLBbGaayzxaaaaaaGccaGLPmIaayPkJaWaaSbaaSqaaiabhkeacnaaBaaameaacqWGYbGCaeqaaaWcbeaakiabg2da9iabicdaWaaa@3EE0@ and 〈ug[k]|ug[l]〉Br=0
 MathType@MTEF@5@5@+=feaafiart1ev1aaatCvAUfKttLearuWrP9MDH5MBPbIqV92AaeXatLxBI9gBaebbnrfifHhDYfgasaacH8akY=wiFfYdH8Gipec8Eeeu0xXdbba9frFj0=OqFfea0dXdd9vqai=hGuQ8kuc9pgc9s8qqaq=dirpe0xb9q8qiLsFr0=vr0=vr0dc8meaabaqaciaacaGaaeqabaqabeGadaaakeaadaaadaqaaiabhwha1naaDaaaleaacqWGNbWzaeaadaWadaqaaiabdUgaRbGaay5waiaaw2faaaaakiabcYha8jabhwha1naaDaaaleaacqWGNbWzaeaadaWadaqaaiabdYgaSbGaay5waiaaw2faaaaaaOGaayzkJiaawQYiamaaBaaaleaacqWHcbGqdaWgaaadbaGaemOCaihabeaaaSqabaGccqGH9aqpcqaIWaamaaa@418A@ for all *k*, *l *(1 ≤ *k *<*l*), and all *g *(1 ≤ *g *≤ *G*).

The value *π*_*g *_is a weight attributed to the triplet (**Y**_*g*_, Iνg
 MathType@MTEF@5@5@+=feaafiart1ev1aaatCvAUfKttLearuWrP9MDH5MBPbIqV92AaeXatLxBI9gBaebbnrfifHhDYfgasaacH8akY=wiFfYdH8Gipec8Eeeu0xXdbba9frFj0=OqFfea0dXdd9vqai=hGuQ8kuc9pgc9s8qqaq=dirpe0xb9q8qiLsFr0=vr0=vr0dc8meaabaqaciaacaGaaeqabaqabeGadaaakeaacqWHjbqsdaWgaaWcbaacciGae8xVd42aaSbaaWqaaiabdEgaNbqabaaaleqaaaaa@3145@, **B**_*r*_) so as to homogenize the impact of each triplet in the multiple analysis. We use *π*_*g *_equal to the inverse of the inertia of the triplet (**Y**_*g*_, Iνg
 MathType@MTEF@5@5@+=feaafiart1ev1aaatCvAUfKttLearuWrP9MDH5MBPbIqV92AaeXatLxBI9gBaebbnrfifHhDYfgasaacH8akY=wiFfYdH8Gipec8Eeeu0xXdbba9frFj0=OqFfea0dXdd9vqai=hGuQ8kuc9pgc9s8qqaq=dirpe0xb9q8qiLsFr0=vr0=vr0dc8meaabaqaciaacaGaaeqabaqabeGadaaakeaacqWHjbqsdaWgaaWcbaacciGae8xVd42aaSbaaWqaaiabdEgaNbqabaaaleqaaaaa@3145@, **B**_*r*_), sum of all its eigenvalues. Let **U**_*g *_be the matrix [ug[1]|...|ug[k]|...|ug[K]]
 MathType@MTEF@5@5@+=feaafiart1ev1aaatCvAUfKttLearuWrP9MDH5MBPbIqV92AaeXatLxBI9gBaebbnrfifHhDYfgasaacH8akY=wiFfYdH8Gipec8Eeeu0xXdbba9frFj0=OqFfea0dXdd9vqai=hGuQ8kuc9pgc9s8qqaq=dirpe0xb9q8qiLsFr0=vr0=vr0dc8meaabaqaciaacaGaaeqabaqabeGadaaakeaadaWadaqaaiabhwha1naaDaaaleaacqWGNbWzaeaadaWadaqaaiabigdaXaGaay5waiaaw2faaaaakiabcYha8jabc6caUiabc6caUiabc6caUiabcYha8jabhwha1naaDaaaleaacqWGNbWzaeaadaWadaqaaiabdUgaRbGaay5waiaaw2faaaaakiabcYha8jabc6caUiabc6caUiabc6caUiabcYha8jabhwha1naaDaaaleaacqWGNbWzaeaadaWadaqaaiabdUealbGaay5waiaaw2faaaaaaOGaay5waiaaw2faaaaa@4C49@ and **V **the matrix [**v**^[1]^|...|**v**^[*k*]^|...|**v**^[*k*]^]. The individual analyses can be projected on the MCoA space. In this space, it is possible to compare the coordinates of the populations according to the consensus of the information provided by the different loci to the coordinates of the populations obtained from each locus. While **V **contains the consensual coordinates of the populations, the coordinates at which the *g*^th ^locus positions the populations are obtained from LYg=πgYgUg
 MathType@MTEF@5@5@+=feaafiart1ev1aaatCvAUfKttLearuWrP9MDH5MBPbIqV92AaeXatLxBI9gBaebbnrfifHhDYfgasaacH8akY=wiFfYdH8Gipec8Eeeu0xXdbba9frFj0=OqFfea0dXdd9vqai=hGuQ8kuc9pgc9s8qqaq=dirpe0xb9q8qiLsFr0=vr0=vr0dc8meaabaqaciaacaGaaeqabaqabeGadaaakeaacqWHmbatdaWgaaWcbaGaemywaK1aaSbaaWqaaiabdEgaNbqabaaaleqaaOGaeyypa0ZaaOaaaeaaiiGacqWFapaCdaWgaaWcbaGaem4zaCgabeaaaeqaaOGaeCywaK1aaSbaaSqaaiabdEgaNbqabaGccqWHvbqvdaWgaaWcbaGaem4zaCgabeaaaaa@3ABE@. Because Yg=Br−1FgtXg
 MathType@MTEF@5@5@+=feaafiart1ev1aaatCvAUfKttLearuWrP9MDH5MBPbIqV92AaeXatLxBI9gBaebbnrfifHhDYfgasaacH8akY=wiFfYdH8Gipec8Eeeu0xXdbba9frFj0=OqFfea0dXdd9vqai=hGuQ8kuc9pgc9s8qqaq=dirpe0xb9q8qiLsFr0=vr0=vr0dc8meaabaqaciaacaGaaeqabaqabeGadaaakeaacqWHzbqwdaWgaaWcbaGaem4zaCgabeaakiabg2da9iabhkeacnaaDaaaleaacqWGYbGCaeaacqGHsislcqaIXaqmaaGccqWHgbGrdaqhaaWcbaGaem4zaCgabaGaemiDaqhaaOGaeCiwaG1aaSbaaSqaaiabdEgaNbqabaaaaa@3BE8@, the matrix LXg=πgXgUg
 MathType@MTEF@5@5@+=feaafiart1ev1aaatCvAUfKttLearuWrP9MDH5MBPbIqV92AaeXatLxBI9gBaebbnrfifHhDYfgasaacH8akY=wiFfYdH8Gipec8Eeeu0xXdbba9frFj0=OqFfea0dXdd9vqai=hGuQ8kuc9pgc9s8qqaq=dirpe0xb9q8qiLsFr0=vr0=vr0dc8meaabaqaciaacaGaaeqabaqabeGadaaakeaacqWHmbatdaWgaaWcbaGaemiwaG1aaSbaaWqaaiabdEgaNbqabaaaleqaaOGaeyypa0ZaaOaaaeaaiiGacqWFapaCdaWgaaWcbaGaem4zaCgabeaaaeqaaOGaeCiwaG1aaSbaaSqaaiabdEgaNbqabaGccqWHvbqvdaWgaaWcbaGaem4zaCgabeaaaaa@3ABA@ positions the alleles of the *g*^th ^locus, so that each population is at the centroid of its allelic composition. However, to compare the individual analyses with the compromise, it is better to **D**_*r*_-normalize LYg
 MathType@MTEF@5@5@+=feaafiart1ev1aaatCvAUfKttLearuWrP9MDH5MBPbIqV92AaeXatLxBI9gBaebbnrfifHhDYfgasaacH8akY=wiFfYdH8Gipec8Eeeu0xXdbba9frFj0=OqFfea0dXdd9vqai=hGuQ8kuc9pgc9s8qqaq=dirpe0xb9q8qiLsFr0=vr0=vr0dc8meaabaqaciaacaGaaeqabaqabeGadaaakeaacqWHmbatdaWgaaWcbaGaemywaK1aaSbaaWqaaiabdEgaNbqabaaaleqaaaaa@30C7@ and LXg
 MathType@MTEF@5@5@+=feaafiart1ev1aaatCvAUfKttLearuWrP9MDH5MBPbIqV92AaeXatLxBI9gBaebbnrfifHhDYfgasaacH8akY=wiFfYdH8Gipec8Eeeu0xXdbba9frFj0=OqFfea0dXdd9vqai=hGuQ8kuc9pgc9s8qqaq=dirpe0xb9q8qiLsFr0=vr0=vr0dc8meaabaqaciaacaGaaeqabaqabeGadaaakeaacqWHmbatdaWgaaWcbaGaemiwaG1aaSbaaWqaaiabdEgaNbqabaaaleqaaaaa@30C5@ because **V **is by definition **D**_*r*_-normalized.

### DPCoA and STATIS

The STATIS analysis applied to (Y1,Iν1,Br),...,(Yg,Iνg,Br),...,(YG,IνG,Br)
 MathType@MTEF@5@5@+=feaafiart1ev1aaatCvAUfKttLearuWrP9MDH5MBPbIqV92AaeXatLxBI9gBaebbnrfifHhDYfgasaacH8akY=wiFfYdH8Gipec8Eeeu0xXdbba9frFj0=OqFfea0dXdd9vqai=hGuQ8kuc9pgc9s8qqaq=dirpe0xb9q8qiLsFr0=vr0=vr0dc8meaabaqaciaacaGaaeqabaqabeGadaaakeaadaqadaqaaiabhMfaznaaBaaaleaacqaIXaqmaeqaaOGaeiilaWIaeCysaK0aaSbaaSqaaGGaciab=17aUnaaBaaameaacqaIXaqmaeqaaaWcbeaakiabcYcaSiabhkeacnaaBaaaleaacqWGYbGCaeqaaaGccaGLOaGaayzkaaGaeiilaWIaeiOla4IaeiOla4IaeiOla4IaeiilaWYaaeWaaeaacqWHzbqwdaWgaaWcbaGaem4zaCgabeaakiabcYcaSiabhMeajnaaBaaaleaacqWF9oGBdaWgaaadbaGaem4zaCgabeaaaSqabaGccqGGSaalcqWHcbGqdaWgaaWcbaGaemOCaihabeaaaOGaayjkaiaawMcaaiabcYcaSiabc6caUiabc6caUiabc6caUiabcYcaSmaabmaabaGaeCywaK1aaSbaaSqaaiabdEeahbqabaGccqGGSaalcqWHjbqsdaWgaaWcbaGae8xVd42aaSbaaWqaaiabdEeahbqabaaaleqaaOGaeiilaWIaeCOqai0aaSbaaSqaaiabdkhaYbqabaaakiaawIcacaGLPaaaaaa@5C62@ implies the calculation of a degree of correlation among the triplets, the so-called *Rν *coefficient. The matrix

Eg=Br1/2YgYgtBr1/2‖Br1/2YgYgtBr1/2‖
 MathType@MTEF@5@5@+=feaafiart1ev1aaatCvAUfKttLearuWrP9MDH5MBPbIqV92AaeXatLxBI9gBaebbnrfifHhDYfgasaacH8akY=wiFfYdH8Gipec8Eeeu0xXdbba9frFj0=OqFfea0dXdd9vqai=hGuQ8kuc9pgc9s8qqaq=dirpe0xb9q8qiLsFr0=vr0=vr0dc8meaabaqaciaacaGaaeqabaqabeGadaaakeaacqWHfbqrdaWgaaWcbaGaem4zaCgabeaakiabg2da9maalaaabaGaeCOqai0aa0baaSqaaiabdkhaYbqaaiabigdaXiabc+caViabikdaYaaakiabhMfaznaaBaaaleaacqWGNbWzaeqaaOGaeCywaK1aa0baaSqaaiabdEgaNbqaaiabdsha0baakiabhkeacnaaDaaaleaacqWGYbGCaeaacqaIXaqmcqGGVaWlcqaIYaGmaaaakeaadaqbdaqaaiabhkeacnaaDaaaleaacqWGYbGCaeaacqaIXaqmcqGGVaWlcqaIYaGmaaGccqWHzbqwdaWgaaWcbaGaem4zaCgabeaakiabhMfaznaaDaaaleaacqWGNbWzaeaacqWG0baDaaGccqWHcbGqdaqhaaWcbaGaemOCaihabaGaeGymaeJaei4la8IaeGOmaidaaaGccaGLjWUaayPcSdaaaaaa@5795@

is at the core of our application of STATIS because it is symmetrical and its dimensions are similar for all the triplets, whereas the dimensions of **Y**_*g *_change. The definition of *Rν *is

Rv(Yg,Yh)=Covv(Yg,Yh)Vav(Yg)Vav(Yh),
 MathType@MTEF@5@5@+=feaafiart1ev1aaatCvAUfKttLearuWrP9MDH5MBPbIqV92AaeXatLxBI9gBaebbnrfifHhDYfgasaacH8akY=wiFfYdH8Gipec8Eeeu0xXdbba9frFj0=OqFfea0dXdd9vqai=hGuQ8kuc9pgc9s8qqaq=dirpe0xb9q8qiLsFr0=vr0=vr0dc8meaabaqaciaacaGaaeqabaqabeGadaaakeaacqWGsbGucqWG2bGDdaqadaqaaiabhMfaznaaBaaaleaacqWGNbWzaeqaaOGaeiilaWIaeCywaK1aaSbaaSqaaiabdIgaObqabaaakiaawIcacaGLPaaacqGH9aqpdaWcaaqaaiabdoeadjabd+gaVjabdAha2jabdAha2naabmaabaGaeCywaK1aaSbaaSqaaiabdEgaNbqabaGccqGGSaalcqWHzbqwdaWgaaWcbaGaemiAaGgabeaaaOGaayjkaiaawMcaaaqaamaakaaabaGaemOvayLaemyyaeMaemODay3aaeWaaeaacqWHzbqwdaWgaaWcbaGaem4zaCgabeaaaOGaayjkaiaawMcaaaWcbeaakmaakaaabaGaemOvayLaemyyaeMaemODay3aaeWaaeaacqWHzbqwdaWgaaWcbaGaemiAaGgabeaaaOGaayjkaiaawMcaaaWcbeaaaaGccqGGSaalaaa@578A@

where

Vav(Yg)=Trace(YgYgtBrYgYgtBr)
 MathType@MTEF@5@5@+=feaafiart1ev1aaatCvAUfKttLearuWrP9MDH5MBPbIqV92AaeXatLxBI9gBaebbnrfifHhDYfgasaacH8akY=wiFfYdH8Gipec8Eeeu0xXdbba9frFj0=OqFfea0dXdd9vqai=hGuQ8kuc9pgc9s8qqaq=dirpe0xb9q8qiLsFr0=vr0=vr0dc8meaabaqaciaacaGaaeqabaqabeGadaaakeaacqWGwbGvcqWGHbqycqWG2bGDdaqadaqaaiabhMfaznaaBaaaleaacqWGNbWzaeqaaaGccaGLOaGaayzkaaGaeyypa0JaemivaqLaemOCaiNaemyyaeMaem4yamMaemyzau2aaeWaaeaacqWHzbqwdaWgaaWcbaGaem4zaCgabeaakiabhMfaznaaDaaaleaacqWGNbWzaeaacqWG0baDaaGccqWHcbGqdaWgaaWcbaGaemOCaihabeaakiabhMfaznaaBaaaleaacqWGNbWzaeqaaOGaeCywaK1aa0baaSqaaiabdEgaNbqaaiabdsha0baakiabhkeacnaaBaaaleaacqWGYbGCaeqaaaGccaGLOaGaayzkaaaaaa@518C@

Covv(Yg,Yh)=Trace(YgYgtBrYhYhtBr)
 MathType@MTEF@5@5@+=feaafiart1ev1aaatCvAUfKttLearuWrP9MDH5MBPbIqV92AaeXatLxBI9gBaebbnrfifHhDYfgasaacH8akY=wiFfYdH8Gipec8Eeeu0xXdbba9frFj0=OqFfea0dXdd9vqai=hGuQ8kuc9pgc9s8qqaq=dirpe0xb9q8qiLsFr0=vr0=vr0dc8meaabaqaciaacaGaaeqabaqabeGadaaakeaacqWGdbWqcqWGVbWBcqWG2bGDcqWG2bGDdaqadaqaaiabhMfaznaaBaaaleaacqWGNbWzaeqaaOGaeiilaWIaeCywaK1aaSbaaSqaaiabdIgaObqabaaakiaawIcacaGLPaaacqGH9aqpcqWGubavcqWGYbGCcqWGHbqycqWGJbWycqWGLbqzdaqadaqaaiabhMfaznaaBaaaleaacqWGNbWzaeqaaOGaeCywaK1aa0baaSqaaiabdEgaNbqaaiabdsha0baakiabhkeacnaaBaaaleaacqWGYbGCaeqaaOGaeCywaK1aaSbaaSqaaiabdIgaObqabaGccqWHzbqwdaqhaaWcbaGaemiAaGgabaGaemiDaqhaaOGaeCOqai0aaSbaaSqaaiabdkhaYbqabaaakiaawIcacaGLPaaaaaa@56A9@

The pairwise calculation of *Rν *leads to a square matrix describing the correlations among the loci. With its eigenvalue decomposition, it is possible to describe the correlation pattern, called the interstructure. Its first eigenvector **α **= (*α*_1_,..., *α*_*g*_,..., *α*_*G*_) is positive and maximizes the quantity ∑g=1G∑h=1GagalRv(Yg,Yh)
 MathType@MTEF@5@5@+=feaafiart1ev1aaatCvAUfKttLearuWrP9MDH5MBPbIqV92AaeXatLxBI9gBaebbnrfifHhDYfgasaacH8akY=wiFfYdH8Gipec8Eeeu0xXdbba9frFj0=OqFfea0dXdd9vqai=hGuQ8kuc9pgc9s8qqaq=dirpe0xb9q8qiLsFr0=vr0=vr0dc8meaabaqaciaacaGaaeqabaqabeGadaaakeaadaaeWaqaamaaqadabaGaemyyae2aaSbaaSqaaiabdEgaNbqabaGccqWGHbqydaWgaaWcbaGaemiBaWgabeaakiabdkfasjabdAha2naabmaabaGaeCywaK1aaSbaaSqaaiabdEgaNbqabaGccqGGSaalcqWHzbqwdaWgaaWcbaGaemiAaGgabeaaaOGaayjkaiaawMcaaaWcbaGaemiAaGMaeyypa0JaeGymaedabaGaem4raCeaniabggHiLdaaleaacqWGNbWzcqGH9aqpcqaIXaqmaeaacqWGhbWra0GaeyyeIuoaaaa@49D9@ where ∑g=1Gag2=1
 MathType@MTEF@5@5@+=feaafiart1ev1aaatCvAUfKttLearuWrP9MDH5MBPbIqV92AaeXatLxBI9gBaebbnrfifHhDYfgasaacH8akY=wiFfYdH8Gipec8Eeeu0xXdbba9frFj0=OqFfea0dXdd9vqai=hGuQ8kuc9pgc9s8qqaq=dirpe0xb9q8qiLsFr0=vr0=vr0dc8meaabaqaciaacaGaaeqabaqabeGadaaakeaadaaeWaqaaiabdggaHnaaDaaaleaacqWGNbWzaeaacqaIYaGmaaGccqGH9aqpcqaIXaqmaSqaaiabdEgaNjabg2da9iabigdaXaqaaiabdEeahbqdcqGHris5aaaa@38D3@. STATIS uses these properties to define a matrix

E=∑g=1GαgBr1/2YgYgtBr1/2‖Br1/2YgYgtBr1/2‖
 MathType@MTEF@5@5@+=feaafiart1ev1aaatCvAUfKttLearuWrP9MDH5MBPbIqV92AaeXatLxBI9gBaebbnrfifHhDYfgasaacH8akY=wiFfYdH8Gipec8Eeeu0xXdbba9frFj0=OqFfea0dXdd9vqai=hGuQ8kuc9pgc9s8qqaq=dirpe0xb9q8qiLsFr0=vr0=vr0dc8meaabaqaciaacaGaaeqabaqabeGadaaakeaacqWHfbqrcqGH9aqpdaaeWaqaaGGaciab=f7aHnaaBaaaleaacqWGNbWzaeqaaOWaaSaaaeaacqWHcbGqdaqhaaWcbaGaemOCaihabaGaeGymaeJaei4la8IaeGOmaidaaOGaeCywaK1aaSbaaSqaaiabdEgaNbqabaGccqWHzbqwdaqhaaWcbaGaem4zaCgabaGaemiDaqhaaOGaeCOqai0aa0baaSqaaiabdkhaYbqaaiabigdaXiabc+caViabikdaYaaaaOqaamaafmaabaGaeCOqai0aa0baaSqaaiabdkhaYbqaaiabigdaXiabc+caViabikdaYaaakiabhMfaznaaBaaaleaacqWGNbWzaeqaaOGaeCywaK1aa0baaSqaaiabdEgaNbqaaiabdsha0baakiabhkeacnaaDaaaleaacqWGYbGCaeaacqaIXaqmcqGGVaWlcqaIYaGmaaaakiaawMa7caGLkWoaaaaaleaacqWGNbWzcqGH9aqpcqaIXaqmaeaacqWGhbWra0GaeyyeIuoaaaa@5FA1@

whose eigenanalysis, **E **= **UΛU**^*t*^, leads to the best compromise of the population pattern over the *G *loci. Note that ‖Br1/2YgYgtBr1/2‖=Vav(Yg)
 MathType@MTEF@5@5@+=feaafiart1ev1aaatCvAUfKttLearuWrP9MDH5MBPbIqV92AaeXatLxBI9gBaebbnrfifHhDYfgasaacH8akY=wiFfYdH8Gipec8Eeeu0xXdbba9frFj0=OqFfea0dXdd9vqai=hGuQ8kuc9pgc9s8qqaq=dirpe0xb9q8qiLsFr0=vr0=vr0dc8meaabaqaciaacaGaaeqabaqabeGadaaakeaadaqbdaqaaiabhkeacnaaDaaaleaacqWGYbGCaeaacqaIXaqmcqGGVaWlcqaIYaGmaaGccqWHzbqwdaWgaaWcbaGaem4zaCgabeaakiabhMfaznaaDaaaleaacqWGNbWzaeaacqWG0baDaaGccqWHcbGqdaqhaaWcbaGaemOCaihabaGaeGymaeJaei4la8IaeGOmaidaaaGccaGLjWUaayPcSdGaeyypa0JaemOvayLaemyyaeMaemODay3aaeWaaeaacqWHzbqwdaWgaaWcbaGaem4zaCgabeaaaOGaayjkaiaawMcaaaaa@4B27@. According to this compromise, the coordinates of the populations are in Br−1/2UΛ1/2
 MathType@MTEF@5@5@+=feaafiart1ev1aaatCvAUfKttLearuWrP9MDH5MBPbIqV92AaeXatLxBI9gBaebbnrfifHhDYfgasaacH8akY=wiFfYdH8Gipec8Eeeu0xXdbba9frFj0=OqFfea0dXdd9vqai=hGuQ8kuc9pgc9s8qqaq=dirpe0xb9q8qiLsFr0=vr0=vr0dc8meaabaqaciaacaGaaeqabaqabeGadaaakeaacqWHcbGqdaqhaaWcbaGaemOCaihabaGaeyOeI0IaeGymaeJaei4la8IaeGOmaidaaOGaeCyvaufcceGae83MdW0aaWbaaSqabeaacqaIXaqmcqGGVaWlcqaIYaGmaaaaaa@38BC@. Owing to Lavit *et al*. [15], the *G *individual population patterns corresponding to the locus considered independently can be obtained. The coordinates of the *i*^th ^populations according to the *g*^th ^locus are the elements of the *i*^th ^row of YgYgtBr1/2UΛ−1/2
 MathType@MTEF@5@5@+=feaafiart1ev1aaatCvAUfKttLearuWrP9MDH5MBPbIqV92AaeXatLxBI9gBaebbnrfifHhDYfgasaacH8akY=wiFfYdH8Gipec8Eeeu0xXdbba9frFj0=OqFfea0dXdd9vqai=hGuQ8kuc9pgc9s8qqaq=dirpe0xb9q8qiLsFr0=vr0=vr0dc8meaabaqaciaacaGaaeqabaqabeGadaaakeaacqWHzbqwdaWgaaWcbaGaem4zaCgabeaakiabhMfaznaaDaaaleaacqWGNbWzaeaacqWG0baDaaGccqWHcbGqdaqhaaWcbaGaemOCaihabaGaeGymaeJaei4la8IaeGOmaidaaOGaeCyvaufcceGae83MdW0aaWbaaSqabeaacqGHsislcqaIXaqmcqGGVaWlcqaIYaGmaaaaaa@3FC6@. Given that Yg=Br−1FgtXg
 MathType@MTEF@5@5@+=feaafiart1ev1aaatCvAUfKttLearuWrP9MDH5MBPbIqV92AaeXatLxBI9gBaebbnrfifHhDYfgasaacH8akY=wiFfYdH8Gipec8Eeeu0xXdbba9frFj0=OqFfea0dXdd9vqai=hGuQ8kuc9pgc9s8qqaq=dirpe0xb9q8qiLsFr0=vr0=vr0dc8meaabaqaciaacaGaaeqabaqabeGadaaakeaacqWHzbqwdaWgaaWcbaGaem4zaCgabeaakiabg2da9iabhkeacnaaDaaaleaacqWGYbGCaeaacqGHsislcqaIXaqmaaGccqWHgbGrdaqhaaWcbaGaem4zaCgabaGaemiDaqhaaOGaeCiwaG1aaSbaaSqaaiabdEgaNbqabaaaaa@3BE8@, the rows of the matrix YgYgtBr1/2UΛ−1/2
 MathType@MTEF@5@5@+=feaafiart1ev1aaatCvAUfKttLearuWrP9MDH5MBPbIqV92AaeXatLxBI9gBaebbnrfifHhDYfgasaacH8akY=wiFfYdH8Gipec8Eeeu0xXdbba9frFj0=OqFfea0dXdd9vqai=hGuQ8kuc9pgc9s8qqaq=dirpe0xb9q8qiLsFr0=vr0=vr0dc8meaabaqaciaacaGaaeqabaqabeGadaaakeaacqWHzbqwdaWgaaWcbaGaem4zaCgabeaakiabhMfaznaaDaaaleaacqWGNbWzaeaacqWG0baDaaGccqWHcbGqdaqhaaWcbaGaemOCaihabaGaeGymaeJaei4la8IaeGOmaidaaOGaeCyvaufcceGae83MdW0aaWbaaSqabeaacqGHsislcqaIXaqmcqGGVaWlcqaIYaGmaaaaaa@3FC6@ position the alleles of the *g*^th ^locus, so that each population is at the centroid of its allelic composition.

### DPCoA and Multiple Factorial Analysis

The MFA is the Principal Component Analysis (PCA) of the global matrix

**Y**_**TOT **_= [*π*_1_**Y**_1_|...|*π*_*g*_**Y**_*g*_|...|*π*_*G*_**Y**_*G*_]:

YTOTtBrYTOT=UΛUt.
 MathType@MTEF@5@5@+=feaafiart1ev1aaatCvAUfKttLearuWrP9MDH5MBPbIqV92AaeXatLxBI9gBaebbnrfifHhDYfgasaacH8akY=wiFfYdH8Gipec8Eeeu0xXdbba9frFj0=OqFfea0dXdd9vqai=hGuQ8kuc9pgc9s8qqaq=dirpe0xb9q8qiLsFr0=vr0=vr0dc8meaabaqaciaacaGaaeqabaqabeGadaaakeaacqWHzbqwdaqhaaWcbaGaeCivaqLaeC4ta8KaeCivaqfabaGaemiDaqhaaOGaeCOqai0aaSbaaSqaaiabdkhaYbqabaGccqWHzbqwdaWgaaWcbaGaeCivaqLaeC4ta8KaeCivaqfabeaakiabg2da9iabhwfavHGabiab=T5amjabhwfavnaaCaaaleqabaGaemiDaqhaaOGaeiOla4caaa@4260@

The global coordinates of the populations synthesizing the information given by all the loci are in **Y**_**TOT**_**U**. The coordinates at which the *g*^th ^locus positions the populations are in

πgYgYgtBrYTOTUΛ−1/2.
 MathType@MTEF@5@5@+=feaafiart1ev1aaatCvAUfKttLearuWrP9MDH5MBPbIqV92AaeXatLxBI9gBaebbnrfifHhDYfgasaacH8akY=wiFfYdH8Gipec8Eeeu0xXdbba9frFj0=OqFfea0dXdd9vqai=hGuQ8kuc9pgc9s8qqaq=dirpe0xb9q8qiLsFr0=vr0=vr0dc8meaabaqaciaacaGaaeqabaqabeGadaaakeaaiiGacqWFapaCdaWgaaWcbaGaem4zaCgabeaakiabhMfaznaaBaaaleaacqWGNbWzaeqaaOGaeCywaK1aa0baaSqaaiabdEgaNbqaaiabdsha0baakiabhkeacnaaBaaaleaacqWGYbGCaeqaaOGaeCywaK1aaSbaaSqaaiabhsfaujabh+eapjabhsfaubqabaGccqWHvbqviiqacqGFBoatdaahaaWcbeqaaiabgkHiTiabigdaXiabc+caViabikdaYaaakiabc6caUaaa@4645@

Because Yg=Br−1FgtXg
 MathType@MTEF@5@5@+=feaafiart1ev1aaatCvAUfKttLearuWrP9MDH5MBPbIqV92AaeXatLxBI9gBaebbnrfifHhDYfgasaacH8akY=wiFfYdH8Gipec8Eeeu0xXdbba9frFj0=OqFfea0dXdd9vqai=hGuQ8kuc9pgc9s8qqaq=dirpe0xb9q8qiLsFr0=vr0=vr0dc8meaabaqaciaacaGaaeqabaqabeGadaaakeaacqWHzbqwdaWgaaWcbaGaem4zaCgabeaakiabg2da9iabhkeacnaaDaaaleaacqWGYbGCaeaacqGHsislcqaIXaqmaaGccqWHgbGrdaqhaaWcbaGaem4zaCgabaGaemiDaqhaaOGaeCiwaG1aaSbaaSqaaiabdEgaNbqabaaaaa@3BE8@, the matrix πgXgYgtBrYTOTUΛ−1/2
 MathType@MTEF@5@5@+=feaafiart1ev1aaatCvAUfKttLearuWrP9MDH5MBPbIqV92AaeXatLxBI9gBaebbnrfifHhDYfgasaacH8akY=wiFfYdH8Gipec8Eeeu0xXdbba9frFj0=OqFfea0dXdd9vqai=hGuQ8kuc9pgc9s8qqaq=dirpe0xb9q8qiLsFr0=vr0=vr0dc8meaabaqaciaacaGaaeqabaqabeGadaaakeaaiiGacqWFapaCdaWgaaWcbaGaem4zaCgabeaakiabhIfaynaaBaaaleaacqWGNbWzaeqaaOGaeCywaK1aa0baaSqaaiabdEgaNbqaaiabdsha0baakiabhkeacnaaBaaaleaacqWGYbGCaeqaaOGaeCywaK1aaSbaaSqaaiabhsfaujabh+eapjabhsfaubqabaGccqWHvbqviiqacqGFBoatdaahaaWcbeqaaiabgkHiTiabigdaXiabc+caViabikdaYaaaaaa@4555@ positions the alleles of the *g*^th ^locus, so that each population is at the centroid of its allelic composition.

### Relationships between the multiple DPCoA and the measurement of diversity

Consider for the two next paragraphs, only one locus – the locus *g*. The DPCoA is centered around a diversity index called "nucleotide diversity" by Nei and Li [23], or "quadratic entropy" by Rao [24], and which is at the core of the Analysis of Molecular Variance (AMOVA) [25-27]:

Hg(pi)=∑k=1ρg∑l=1ρgpkiplidklall,g=pitDall,gpi
 MathType@MTEF@5@5@+=feaafiart1ev1aaatCvAUfKttLearuWrP9MDH5MBPbIqV92AaeXatLxBI9gBaebbnrfifHhDYfgasaacH8akY=wiFfYdH8Gipec8Eeeu0xXdbba9frFj0=OqFfea0dXdd9vqai=hGuQ8kuc9pgc9s8qqaq=dirpe0xb9q8qiLsFr0=vr0=vr0dc8meaabaqaciaacaGaaeqabaqabeGadaaakeaacqWGibasdaWgaaWcbaGaem4zaCgabeaakmaabmaabaGaeCiCaa3aaSbaaSqaaiabdMgaPbqabaaakiaawIcacaGLPaaacqGH9aqpdaaeWbqaamaaqahabaGaeCiCaa3aaSbaaSqaaiabdUgaRjabdMgaPbqabaGccqWHWbaCdaWgaaWcbaGaemiBaWMaemyAaKgabeaakiabdsgaKnaaDaaaleaacqWGRbWAcqWGSbaBaeaacqqGHbqycqqGSbaBcqqGSbaBcqGGSaalcqWGNbWzaaaabaGaemiBaWMaeyypa0JaeGymaedabaacciGae8xWdi3aaSbaaWqaaiabdEgaNbqabaaaniabggHiLdaaleaacqWGRbWAcqGH9aqpcqaIXaqmaeaacqWFbpGCdaWgaaadbaGaem4zaCgabeaaa0GaeyyeIuoakiabg2da9iabhchaWnaaDaaaleaacqWGPbqAaeaacqWG0baDaaGccqWHebardaahaaWcbeqaaiabdggaHjabdYgaSjabdYgaSjabcYcaSiabdEgaNbaakiabhchaWnaaBaaaleaacqWGPbqAaeqaaaaa@69CA@

In this formula, *g *designates the *g*^th ^locus, *ρ*_*g *_is the number of different alleles observed for that locus, pi=(p1i,...,pki,...,pρgi)t
 MathType@MTEF@5@5@+=feaafiart1ev1aaatCvAUfKttLearuWrP9MDH5MBPbIqV92AaeXatLxBI9gBaebbnrfifHhDYfgasaacH8akY=wiFfYdH8Gipec8Eeeu0xXdbba9frFj0=OqFfea0dXdd9vqai=hGuQ8kuc9pgc9s8qqaq=dirpe0xb9q8qiLsFr0=vr0=vr0dc8meaabaqaciaacaGaaeqabaqabeGadaaakeaacqWHWbaCdaWgaaWcbaGaemyAaKgabeaakiabg2da9maabmaabaGaemiCaa3aaSbaaSqaaiabigdaXiabdMgaPbqabaGccqGGSaalcqGGUaGlcqGGUaGlcqGGUaGlcqGGSaalcqWGWbaCdaWgaaWcbaGaem4AaSMaemyAaKgabeaakiabcYcaSiabc6caUiabc6caUiabc6caUiabcYcaSiabdchaWnaaBaaaleaaiiGacqWFbpGCdaWgaaadbaGaem4zaCgabeaaliabdMgaPbqabaaakiaawIcacaGLPaaadaahaaWcbeqaaiabdsha0baaaaa@4B42@ is the vector containing the relative frequencies of the alleles in the *i*^th ^population, so that *p*_*ki *_is the frequency of the allele *k *in the *i*^th ^population, and dklall,g
 MathType@MTEF@5@5@+=feaafiart1ev1aaatCvAUfKttLearuWrP9MDH5MBPbIqV92AaeXatLxBI9gBaebbnrfifHhDYfgasaacH8akY=wiFfYdH8Gipec8Eeeu0xXdbba9frFj0=OqFfea0dXdd9vqai=hGuQ8kuc9pgc9s8qqaq=dirpe0xb9q8qiLsFr0=vr0=vr0dc8meaabaqaciaacaGaaeqabaqabeGadaaakeaacqWGKbazdaqhaaWcbaGaem4AaSMaemiBaWgabaGaeeyyaeMaeeiBaWMaeeiBaWMaeiilaWIaem4zaCgaaaaa@3728@ is the distance among the alleles *k *and *l *of the *g*^th ^locus. The DPCoA uses a decomposition of this diversity component defined by Rao [27]:

*H*_TOTAL, *g*_({*μ*_*i*_},{**p**_*i*_}) = *H*_INTRA, *g*_({*μ*_*i*_},{**p**_*i*_}) + *H*_INTRA, *g*_({*μ*_*i*_},{**p**_*i*_}),

where

HTOTAL,g({μi},{pi})=Hg(∑i=1rμipi),
 MathType@MTEF@5@5@+=feaafiart1ev1aaatCvAUfKttLearuWrP9MDH5MBPbIqV92AaeXatLxBI9gBaebbnrfifHhDYfgasaacH8akY=wiFfYdH8Gipec8Eeeu0xXdbba9frFj0=OqFfea0dXdd9vqai=hGuQ8kuc9pgc9s8qqaq=dirpe0xb9q8qiLsFr0=vr0=vr0dc8meaabaqaciaacaGaaeqabaqabeGadaaakeaacqWGibasdaWgaaWcbaGaeeivaqLaee4ta8KaeeivaqLaeeyqaeKaeeitaWKaeiilaWIaem4zaCgabeaakmaabmaabaWaaiWaaeaaiiGacqWF8oqBdaWgaaWcbaGaemyAaKgabeaaaOGaay5Eaiaaw2haaiabcYcaSmaacmaabaGaeCiCaa3aaSbaaSqaaiabdMgaPbqabaaakiaawUhacaGL9baaaiaawIcacaGLPaaacqGH9aqpcqWGibasdaWgaaWcbaGaem4zaCgabeaakmaabmaabaWaaabCaeaacqWF8oqBdaWgaaWcbaGaemyAaKgabeaakiabhchaWnaaBaaaleaacqWGPbqAaeqaaaqaaiabdMgaPjabg2da9iabigdaXaqaaiabdkhaYbqdcqGHris5aaGccaGLOaGaayzkaaGaeiilaWcaaa@563E@

HINTRA,g({μi},{pi})=∑i=1rμiHg(pi),
 MathType@MTEF@5@5@+=feaafiart1ev1aaatCvAUfKttLearuWrP9MDH5MBPbIqV92AaeXatLxBI9gBaebbnrfifHhDYfgasaacH8akY=wiFfYdH8Gipec8Eeeu0xXdbba9frFj0=OqFfea0dXdd9vqai=hGuQ8kuc9pgc9s8qqaq=dirpe0xb9q8qiLsFr0=vr0=vr0dc8meaabaqaciaacaGaaeqabaqabeGadaaakeaacqWGibasdaWgaaWcbaGaeeysaKKaeeOta4KaeeivaqLaeeOuaiLaeeyqaeKaeiilaWIaem4zaCgabeaakmaabmaabaWaaiWaaeaaiiGacqWF8oqBdaWgaaWcbaGaemyAaKgabeaaaOGaay5Eaiaaw2haaiabcYcaSmaacmaabaGaeCiCaa3aaSbaaSqaaiabdMgaPbqabaaakiaawUhacaGL9baaaiaawIcacaGLPaaacqGH9aqpdaaeWbqaaiab=X7aTnaaBaaaleaacqWGPbqAaeqaaaqaaiabdMgaPjabg2da9iabigdaXaqaaiabdkhaYbqdcqGHris5aOGaemisaG0aaSbaaSqaaiabdEgaNbqabaGcdaqadaqaaiabhchaWnaaBaaaleaacqWGPbqAaeqaaaGccaGLOaGaayzkaaGaeiilaWcaaa@5632@

and

HINTER,g({μi}:{pi})=∑i=1r∑j=1rμiμjdpop,g(pi,pj),
 MathType@MTEF@5@5@+=feaafiart1ev1aaatCvAUfKttLearuWrP9MDH5MBPbIqV92AaeXatLxBI9gBaebbnrfifHhDYfgasaacH8akY=wiFfYdH8Gipec8Eeeu0xXdbba9frFj0=OqFfea0dXdd9vqai=hGuQ8kuc9pgc9s8qqaq=dirpe0xb9q8qiLsFr0=vr0=vr0dc8meaabaqaciaacaGaaeqabaqabeGadaaakeaacqWGibasdaWgaaWcbaGaeeysaKKaeeOta4KaeeivaqLaeeyrauKaeeOuaiLaeiilaWIaem4zaCgabeaakmaabmaabaWaaiWaaeaaiiGacqWF8oqBdaWgaaWcbaGaemyAaKgabeaaaOGaay5Eaiaaw2haaiabcQda6maacmaabaGaeCiCaa3aaSbaaSqaaiabdMgaPbqabaaakiaawUhacaGL9baaaiaawIcacaGLPaaacqGH9aqpdaaeWbqaamaaqahabaGae8hVd02aaSbaaSqaaiabdMgaPbqabaGccqWF8oqBdaWgaaWcbaGaemOAaOgabeaakiabdsgaKnaaCaaaleqabaGaeeiCaaNaee4Ba8MaeeiCaaNaeiilaWIaem4zaCgaaOWaaeWaaeaacqWHWbaCdaWgaaWcbaGaemyAaKgabeaakiabcYcaSiabhchaWnaaBaaaleaacqWGQbGAaeqaaaGccaGLOaGaayzkaaaaleaacqWGQbGAcqGH9aqpcqaIXaqmaeaacqWGYbGCa0GaeyyeIuoaaSqaaiabdMgaPjabg2da9iabigdaXaqaaiabdkhaYbqdcqGHris5aOGaeiilaWcaaa@69DD@

where dpop,g(pi,pj)=2Hg(pi+pj2)−Hg(pi)−Hg(pj)
 MathType@MTEF@5@5@+=feaafiart1ev1aaatCvAUfKttLearuWrP9MDH5MBPbIqV92AaeXatLxBI9gBaebbnrfifHhDYfgasaacH8akY=wiFfYdH8Gipec8Eeeu0xXdbba9frFj0=OqFfea0dXdd9vqai=hGuQ8kuc9pgc9s8qqaq=dirpe0xb9q8qiLsFr0=vr0=vr0dc8meaabaqaciaacaGaaeqabaqabeGadaaakeaacqWGKbazdaahaaWcbeqaaiabbchaWjabb+gaVjabbchaWjabcYcaSiabdEgaNbaakmaabmaabaGaeCiCaa3aaSbaaSqaaiabdMgaPbqabaGccqGGSaalcqWHWbaCdaWgaaWcbaGaemOAaOgabeaaaOGaayjkaiaawMcaaiabg2da9iabikdaYiabdIeainaaBaaaleaacqWGNbWzaeqaaOWaaeWaaeaadaWcaaqaaiabhchaWnaaBaaaleaacqWGPbqAaeqaaOGaey4kaSIaeCiCaa3aaSbaaSqaaiabdQgaQbqabaaakeaacqaIYaGmaaaacaGLOaGaayzkaaGaeyOeI0IaemisaG0aaSbaaSqaaiabdEgaNbqabaGcdaqadaqaaiabhchaWnaaBaaaleaacqWGPbqAaeqaaaGccaGLOaGaayzkaaGaeyOeI0IaemisaG0aaSbaaSqaaiabdEgaNbqabaGcdaqadaqaaiabhchaWnaaBaaaleaacqWGQbGAaeqaaaGccaGLOaGaayzkaaaaaa@5B44@.

In the first step of the DPCoA, all the points (*i.e*. alleles and populations) are in a space called "common space" [11]. In this common space, the inertia (*i.e*. variance) of the allele points weighted by **p**_*i *_is equal to *H*_*g*_(**p**_*i*_), the diversity of the population *i*, according to locus *g*. The inertia of all the allele points weighted by ∑i=1rμipi
 MathType@MTEF@5@5@+=feaafiart1ev1aaatCvAUfKttLearuWrP9MDH5MBPbIqV92AaeXatLxBI9gBaebbnrfifHhDYfgasaacH8akY=wiFfYdH8Gipec8Eeeu0xXdbba9frFj0=OqFfea0dXdd9vqai=hGuQ8kuc9pgc9s8qqaq=dirpe0xb9q8qiLsFr0=vr0=vr0dc8meaabaqaciaacaGaaeqabaqabeGadaaakeaadaaeWaqaaGGaciab=X7aTnaaBaaaleaacqWGPbqAaeqaaOGaeCiCaa3aaSbaaSqaaiabdMgaPbqabaaabaGaemyAaKMaeyypa0JaeGymaedabaGaemOCaihaniabggHiLdaaaa@39A3@ is equal to *H*_TOTAL, *g*_, the total diversity of the data set. Finally, the inertia of all the population points weighted by **μ **= (*μ*_1_,..., *μ*_*i*_,..., *μ*_*r*_) is equal to *H*_INTER, *g*_, the component of diversity among populations [11]. At the end of the DPCoA analysis, all the points are projected in a subspace which optimizes the representation of the differences among populations. In this subspace, only *H*_INTER, *g *_is maintained, which is thus the focus of the analysis: optimally displaying the diversity among populations.

Consequently, the multiple DPCoA allows us to optimize the description of diversity among populations obtained with several loci. The first goal of this method is to describe the differences in population patterns across the loci, hence studying the congruence among loci. Another objective may be to erase these differences and provide a compromise population pattern revealed by the majority of the loci. The DPCoA-STATIS is advocated for this purpose. Concerning the measurement of diversity, when several loci are considered to measure diversity, the sum or average of the diversity components over the loci is currently used as a global measure of diversity [see for example [28,29]]. With such processes, the weights given to the loci for the sum or averaging are uniform. We have just shown that STATIS provides optimal locus weights for the calculation of the component of diversity among populations. The great advantage of these multivariate analyses is that visualization of the differences among loci is possible so that one can assess the relevance of using average information over loci, whether these means are weighted or not.

### Associated tests

We performed both Mantel and *Rν *tests to evaluate the significance of the differences in population patterns among loci. For each locus, distances among populations are calculated with the inter-population diversity *H*_INTER, *g*_({*μ*_*i*_}:{**p**_*i*_}) according to Nei and Li [23] and Rao [24,27]. We just said that this statistic is at the core of the DPCoA. As we apply formula (*H*_INTER, *g*_) in a pairwise fashion, the distance between population *i *and population *j *for locus *g *is *μ*_*i*_*μ*_*j*_*d*^pop, *g*^(**p**_*i*_, **p**_*j*_). We choose *μ*_*i*_*μ*_*j*_*d*^pop, *g*^(**p**_*i*_, **p**_*j*_) and not simply *d*^pop, *g*^(**p**_*i*_, **p**_*j*_) to take into account differential sample sizes, exactly in the way that we considered them in ordination procedures. The Mantel test calculates correlations among the raw distance measures, while the *Rν *test compares principal coordinates obtained by PCoA. *Rν *correlations are always higher than Mantel correlations because their values lie between 0 and 1, while Mantel correlation values lie between -1 and 1.

### Application to simulated and real data sets

We used the following procedure to test the methodologies presented above based on simulated and real data sets. First, pairwise correlations among loci by Mantel and/or *Rν *tests were assessed to define groups of consistent loci. At this step, atypical loci can be identified. Then mDPCoA was performed to describe both the compromise population structure and the differences among groups of loci. Finally, we describe the connections between the observed structures and ecological, evolutionary or functional data.

### Application to a simulated data set

#### Simulation process

In order to assess the efficiency of the present method, simulated sequence data sets, which illustrate various population structures, were obtained assuming linkage equilibrium among loci. Assuming recombination, the different markers can indeed have different histories and thus different population structures. Moreover, if every marker has an independent history, finding similarities and differences among their genetic structures would be more difficult. Using SIMCOAL 2.0 [30] we considered a one-dimensional stepping stone model with eight populations of constant size [31]. The eight populations evolved 10^6 ^generations after emerging from a single ancestral population. For each population, 60 individuals were sampled out of 10000 individuals. In this context, we simulated DNA sequence evolution of ten loci of 300 base pairs under a Jukes and Cantor model [32] assuming a mutation rate of 5 × 10^-6^. The stepping stone model allows migration between adjacent populations: for example, at time t, the population 4 can exchange individuals with populations 3 or 5, but not with other populations. We chose the following migration rates: 5 × 10^-2^, 10^-2^, 5 × 10^-3^, 10^-3^, 5 × 10^-4^, 10^-4^, 5 × 10^-5^, 10^-5^, 5 × 10^-6^. We also simulated an eleventh locus that reveals a different population structure. For this locus, we assumed no migration between odd populations (*i.e*. populations 1, 3, 5, 7) and even populations (*i.e*. populations 2, 4, 6, 8) and a migration rate of 10^-3 ^among odd or even populations, with other parameters kept unchanged. Such a simulation resulted in two clades of alleles which are obviously divergent, the first clade being specific to some populations (e.g. odd ones), the second clade being specific to other populations (e.g. even ones). Such genetic structure can be observed in case of either balancing/disruptive selection [e.g. [33]] or horizontal transfer of an outlier allele [e.g. [7]].

We applied the mDPCoA approach first on the complete data set, second on the allele distances only and then taking into account just the allele frequencies. We evaluated the intensity of inter-population structure by measuring the AMOVA *ϕ*_*ST *_parameter [25].

#### Results

**Figure 1:**
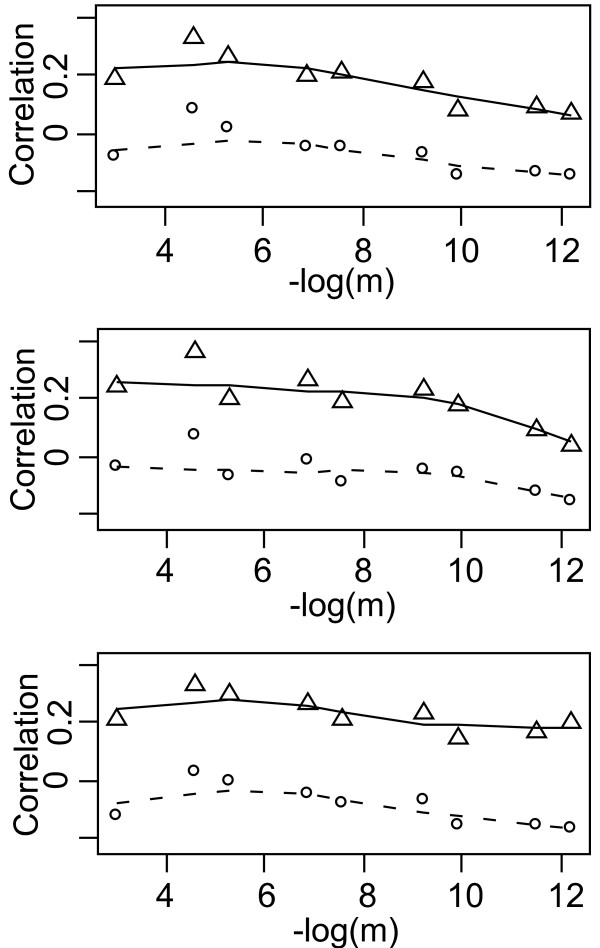
**Mantel and *Rv *correlations between atypical and other loci in the simulated data set**. The parameter *m *is the migration rate of the simulated linear stepping stone. Each statistic is calculated and averaged between the atypical locus and the first 10 loci submitted to a stepping stone model, A) with both allele frequency and distance information, B) with allele distances without allele frequencies, C) with allele frequencies without allele distances. Plain lines with triangle-shaped symbols mark the average *Rν *correlation values, while the broken lines with open circles indicate the average Mantel correlation values.

The correlations among locus 11 and the ten other loci are very low and not significant as expected (Figure 1). Thus, we correctly identified the atypical locus. These correlations decrease when migration rate decreases. Test statistics based on both the Mantel correlation and the *Rν *correlation between the atypical locus and other loci clearly behave in a similar way, and results are hardly changed when removing allele frequencies or distances.

**Figure 2:**
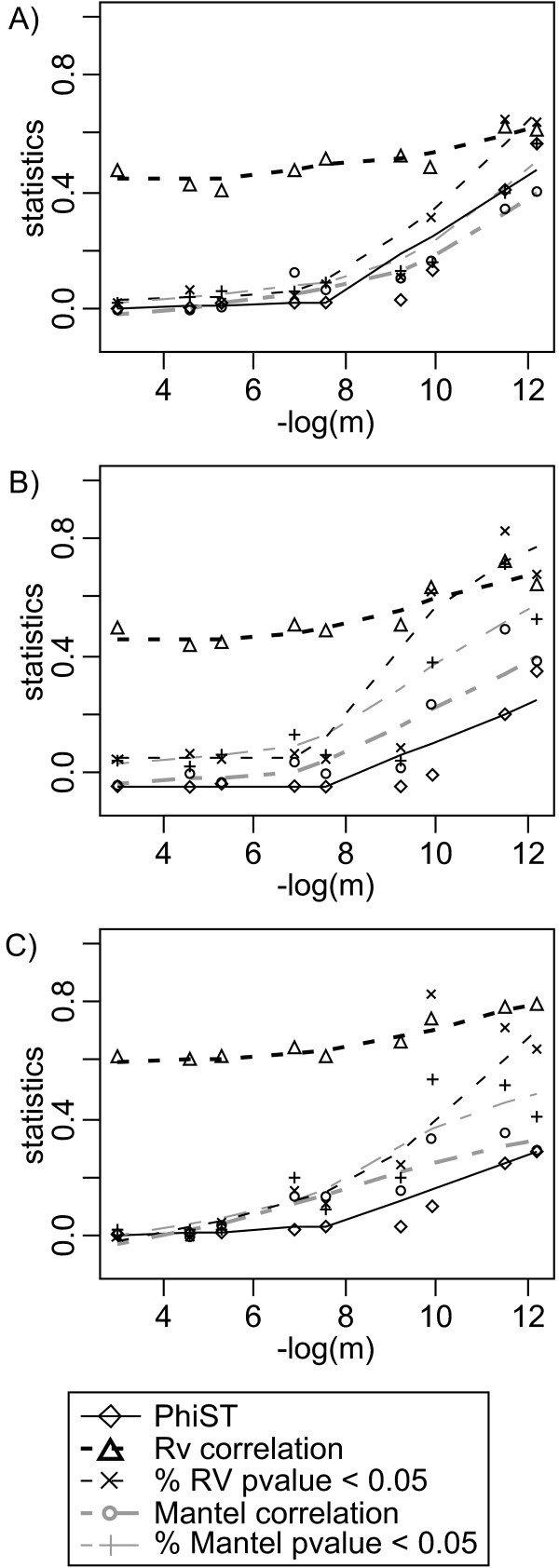
**Mantel and *Rv *correlations among the ten first loci in the simulated data set**. The parameter *m *is the migration rate of the simulated linear stepping stone. Each statistic is calculated on 10 loci submitted to this stepping stone model, A) with allele frequency and distance information, B) with allele distances without allele frequencies, C) with allele frequencies without allele distances. Symbol legends are given at the bottom of the graphs.

Regarding the correlation tests among the 10 loci submitted to the stepping stone model, the inter-population structure measured by the AMOVA *ϕ*_*ST *_parameter increases slightly when the migration rate decreases from 5 × 10^-2 ^to 5 × 10^-4 ^and then increases very quickly (Figure 2). Values of the Mantel correlation, the percent of significant tests according to the Mantel correlation and the percent of significant tests according to the *Rν *correlation are three parameters correlated with *ϕ*_*ST*_, especially when using both allele frequency and allele divergences. The raw value of the *Rν *correlation is steadier. These results show that a non-significant correlation may be due to either an absence of genetic structure (e.g. no differentiation among populations) or reliable differences in the inter-population structures revealed by the different loci. The graphical analysis completed by *ϕ*_*ST *_values will help to reach a conclusion between the two alternatives.

**Figure 3:**
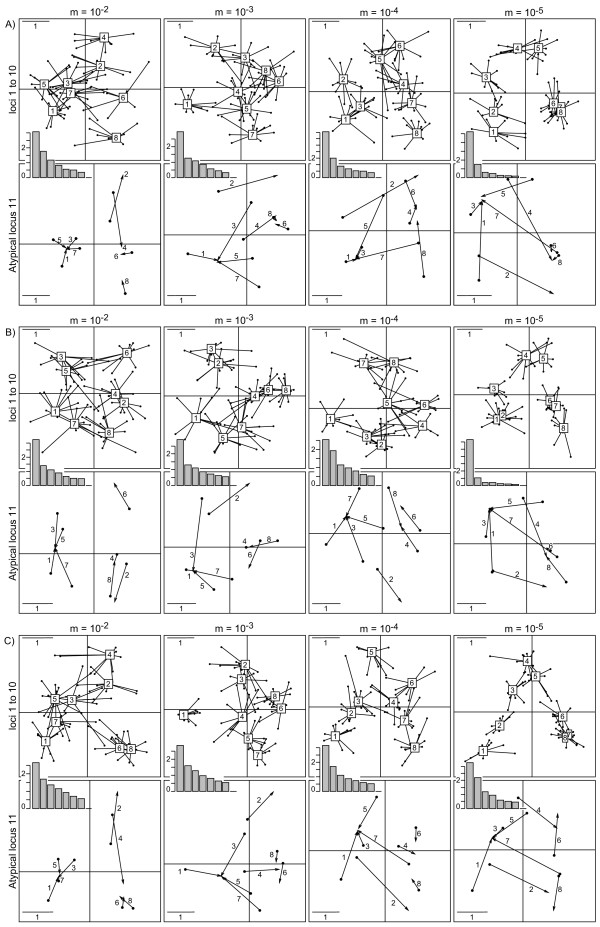
**Application of the DPCoA-MCoA to the simulateddata set**. The parameter *m *is the migration rate of the simulated linear stepping stone. The DPCoA-MCoA was applied on the simulated data set, A) with allele frequency and distance information, B) with allele distances without allele frequencies, C) with allele frequencies without allele distances. Each figure A) B) and C) comprises two series of four subfigures. In the first row, for each locus the compromise pattern of differences among populations (Numbers in boxes) is given with lines relating the compromise to the ten first loci submitted to the stepping stone model. In the second row, for each locus the compromise pattern of population differences is also given at the beginning of the arrows, and this time, the arrows point at the position of each population according to the atypical locus. The longer the arrow, the more different the pattern inferred by the atypical locus from the compromise pattern. Eigenvalue barplots are provided for analyses A), B), and C).

Regarding the mDPCoA, we present below the results of the DPCoA-MCoA approach, which we expected to provide a description of the difference among the ten first loci and the eleventh, atypical locus (Figure 3; to limit the size of the Figure 3, only the results for migration rates 10^-2^, 10^-3^, 10^-4 ^and 10^-5 ^are shown since intermediate migration rates revealed intermediate inter-population structure). Indeed, for migration rates higher than 10^-2^, where no inter-population structure was highlighted in the previous paragraph, the atypical locus takes the first axis of the compromise analysis, which therefore distinguishes odd from even populations. With a migration rate of 10^-3^, the stepping stone model interacts with the structure provided by locus 11; the 10 first loci with a stepping stone model take the first axis and locus 11 roughly takes the second axis. With a migration rate lower than 10^-3^, the first two axes of the DPCoA-MCoA only represent the stepping stone model. Whatever the migration rate, the projection of the individual loci on the DPCoA-MCoA factorial axes emphasizes locus 11's special status (Figure 3). This last result is also emphasized by specific results of the DPCoA-STATIS approach as interstructures. With a migration rate equal to 5 × 10^-4 ^or lower, the structure is very clear with either complete or incomplete data on allele composition.

### Application to the description of Sinorhizobium species diversity

#### The data set

In order to test the efficiency of the procedures we proposed, we needed a real data set which should give simple and explicit results but which could also encompass the features of complex MLS data sets. We chose to focus on nitrogen fixing bacteria belonging to the genus *Sinorhizobium *(Rhizobiaceae) associated with the plant genus *Medicago *(Fabaceae). The data set we chose is a combination of two data sets fully available online from GenBank and published in two recent papers [8,34]. The complete sampling procedure is described in the two papers and summarized in an additional file [see Additional file 3]. Based on the sampling scheme, we delineated six populations according to geographical origin (France: F, Tunisia Hadjeb: TH, Tunisia Enfidha: TE), the host plant (*M. truncatula *or similar symbiotic specificity: T, *M. laciniata*: L), and the taxonomical status of bacteria (*S. meliloti*: mlt, *S. medicae*: mdc). Each population will be called hereafter according to the three above criteria, *e.g*. THLmlt is the population sampled in Tunisia at Hadjeb from *M. laciniata *nodules which include *S. meliloti *isolates. *S. medicae *interacts with *M. truncatula *while *S. meliloti *interacts with both *M. laciniata *(*S. meliloti *bv. medicaginis) and *M. truncatula *(*S. meliloti *bv. meliloti) [35,36]. The numbers of individuals are respectively 46 for FTmdc, 43 for FTmlt, 20 for TETmdc, 24 for TETmlt, 20 for TELmlt, 42 for THTmlt and 20 for THLmlt [see Additional files 4, 5, 6, 7].

**Figure 4:**
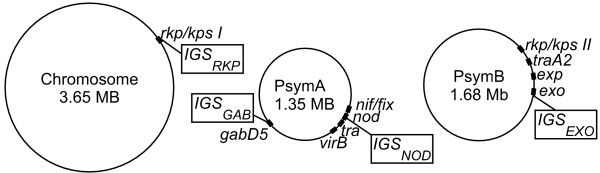
**Location of genetic markers on the genome of *Sinorhizobium meliloti *strain 1021**. Gene clusters located nearby each genetic marker are indicated by black boxes. It is noteworthy that the *IGS*_*NOD *_marker is located near genes involved in symbiotic specificity (*nod *genes), symbiotic efficiency (*nif*/*fix *genes), secretion (*virB *gene) and conjugation (*tra *genes). *IGS*_*RKP *_and *IGS*_*EXO *_are located near genes involved in the synthesis of surface polysaccharides, which are also involved in the symbiotic interaction. *IGS*_*GAB *_is physically close to genes involved in secondary metabolic pathways.

Four different intergenic spacers (*IGS*), *IGS*_*NOD*_, *IGS*_*EXO*_, *IGS*_*GAB*, _and *IGS*_*RKP*_, distributed on the different replication units of the model strain 1021 of *S. meliloti *bv. meliloti (Figure 4) had been sequenced to characterize each bacterial isolate (DNA extraction and sequencing procedures are described in an additional file [see Additional file 3]). It is noteworthy that the *IGS*_*NOD *_marker is located within the *nod *gene cluster and that specific alleles at these loci determine the ability of *S. meliloti *strains to interact with either *M. laciniata *or *M. truncatula *[37].

**Figure 5:**
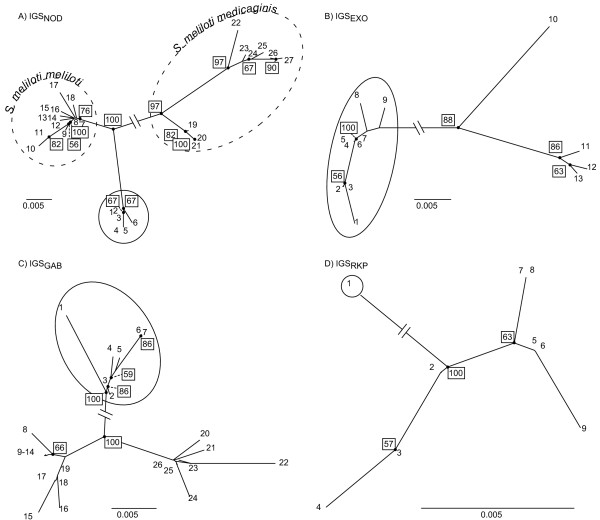
**Neighbor-Joining trees for the representation of the distances among alleles**. The alleles belonging to *S. medicae *isolates are surrounded by a plain-line circle. Only *IGS*_*NOD *_presents alleles found only in *S. meliloti *bv. meliloti populations and alleles found only in *S. meliloti *bv. medicaginis. Consequently, for *IGS*_*NOD*_, alleles are also divided according the two biovars of *S. meliloti*, by broken-line circles. Bootstrap values higher than 50% are given in boxes. Nodes with bootstrap values higher than 50% are indicated by plain circles and in case of possible ambiguity, a broken line links the node to the bootstrap value. The interrupted lines have a length of 0.0986 for *IGS*_*NOD*_, 0.1075 for *IGS*_*EXO*_, 0.0456 for *IGS*_*GAB *_and 0.0421 for *IGS*_*RKP*_.

For each locus, we selected a model of evolution using the software PHYML [38] and its R interface provided by ape [18,19]. This software compares the models by likelihood ratio tests. When several models were not significantly different according to a χ^2 ^test we selected the model with the smallest number of parameters. From this procedure, we selected Felsenstein's model F84 [39,40] for *IGS*_*NOD*_, *IGS*_*EXO*_, *IGS*_*GAB*_, and Felsenstein's model F81 [40,41] for *IGS*_*RKP*_. Then, using the ape package, a set of matrices {DIGSNOD,DIGSEXO,DIGSGAB,DIGSRKP}
 MathType@MTEF@5@5@+=feaafiart1ev1aaatCvAUfKttLearuWrP9MDH5MBPbIqV92AaeXatLxBI9gBaebbnrfifHhDYfgasaacH8akY=wiFfYdH8Gipec8Eeeu0xXdbba9frFj0=OqFfea0dXdd9vqai=hGuQ8kuc9pgc9s8qqaq=dirpe0xb9q8qiLsFr0=vr0=vr0dc8meaabaqaciaacaGaaeqabaqabeGadaaakeaadaGadaqaaiabhseaenaaBaaaleaacqWGjbqscqWGhbWrcqWGtbWudaWgaaadbaGaemOta4Kaem4ta8KaemiraqeabeaaaSqabaGccqGGSaalcqWHebardaWgaaWcbaGaemysaKKaem4raCKaem4uam1aaSbaaWqaaiabdweafjabdIfayjabd+eapbqabaaaleqaaOGaeiilaWIaeCiraq0aaSbaaSqaaiabdMeajjabdEeahjabdofatnaaBaaameaacqWGhbWrcqWGbbqqcqWGcbGqaeqaaaWcbeaakiabcYcaSiabhseaenaaBaaaleaacqWGjbqscqWGhbWrcqWGtbWudaWgaaadbaGaemOuaiLaem4saSKaemiuaafabeaaaSqabaaakiaawUhacaGL9baaaaa@5281@ containing pairwise genetic distances between alleles observed at each locus was computed according to these selected models, and Neighbor-Joining trees with bootstrap values were obtained from these distance matrices to illustrate the data sets (Figure 5).

We applied the multiple DPCoA to this data set, and compared the results to those obtained with STRUCTURE [42,43]. STRUCTURE estimates population structure using genotype data. The basic hypotheses are linkage equilibrium within subpopulations (or possibly weak linkage [44]) and Hardy-Weinberg equilibrium (if the organism under study is not haploid).

#### Results

Mantel and *Rν *tests demonstrated that the locus *IGS*_*NOD *_provides a very specific ordination of populations, while the three other markers *IGS*_*RKP*_, *IGS*_*EXO *_and *IGS*_*GAB*_, were significantly congruent (Table 1).

**Table 1:** Pairwise correlations among loci with the complete real data set

Mantel	*IGS*_*NOD*_	*IGS*_*EXO*_	*IGS*_*GAB*_	Rv tests	*IGS*_*NOD*_	*IGS*_*EXO*_	*IGS*_*GAB*_
*IGS*_*EXO*_	-0.164			*IGS*_*EXO*_	0.232		
*IGS*_*GAB*_	-0.173	1.000*		*IGS*_*GAB*_	0.230	1.000*	
*IGS*_*RKP*_	-0.164	1.000*	0.999*	*IGS*_*RKP*_	0.227	1.000*	0.999*

*Significant correlations with P-values < 0.05.

**Figure 6:**
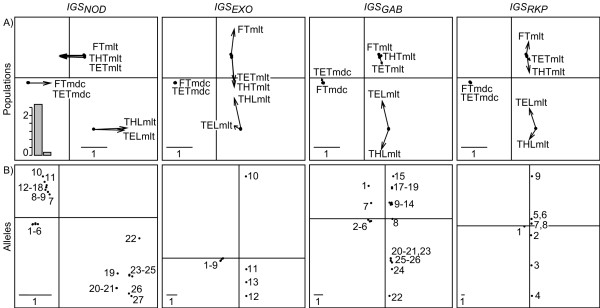
**Application of the DPCoA-MCoA to the real data set**. A) Comparison between the patterns of the differences among populations given by the compromise over all loci (black dots, start of the arrows) and the individual analyses (end of the arrows). The special status of *IGS*_*NOD *_is highlighted by horizontal arrows (wrong assignment on the first axis), whereas *IGS*_*GAB*_, *IGS*_*RKP *_and *IGS*_*EXO *_presents vertical arrows (discrepancies from the compromise structure on axis 2 only); B) Location of the alleles. A low (or high) variance in allele points on an axis indicates that the diversity among alleles within populations is lower (or higher) than the diversity among populations, because each axis is normalized for diversity among populations. An eigenvalue barplot is provided in the left-hand corner.

**Figure 7:**
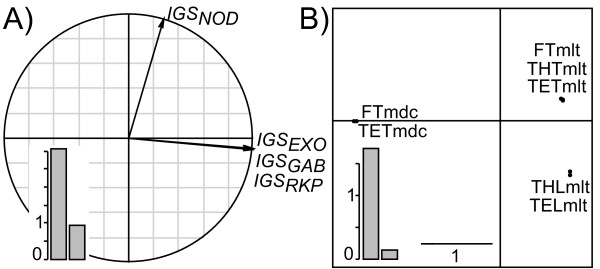
**Application of the DPCoA-STATIS to the real data set**. A) The interstructure which displays the eigenanalysis of the *Rν *matrix, and B) the best compromise. Eigenvalue barplots are provided in boxes. In the interstructure (A), the smaller the angle between two loci, the more similar the inter-population patterns provided by the two loci.

With DPCoA-MCoA (Figure 6), the first axis, which expresses 94% of the diversity among populations, separates the two bacterial species, *S. meliloti *and *S. medicae*, while the second axis, with 6% of the diversity among populations, distinguishes the impact of the host plants, *M. laciniata *and *M. truncatula*. The DPCoA-STATIS analysis reveals a very similar pattern (Figure 7). Consistently, the STRUCTURE analysis indeed defined two main clusters including respectively *S. meliloti *and *S. medicae*, without any trace of admixture between the two species. However, these results are a compromise with the information provided by *IGS*_*RKP*_, *IGS*_*GAB*_, *IGS*_*EXO *_and *IGS*_*NOD*_. Although the four markers effectively delineate the two bacterial species, they express this segregation differently. The DPCoA-MCoA indeed revealed that the segregation between *S. meliloti *and *S. medicae *is supported by more than 90% population variation for the three most coherent markers, *i.e. IGS*_*RKP*_, *IGS*_*GAB *_and *IGS*_*EXO*_, while it only concerns a minor part of the population variation observed for *IGS*_*NOD*_. The discrimination between the impact of the two host plants, *i.e. M. truncatula *and *M. laciniata*, which appears in axis 2, is the main structure for the *IGS*_*NOD *_marker. The interstructure obtained by using STATIS (Figure 7A), *i.e*. the eigenanalysis of the *Rν *matrix, illustrated the special status of *IGS*_*NOD*_.

**Figure 8:**
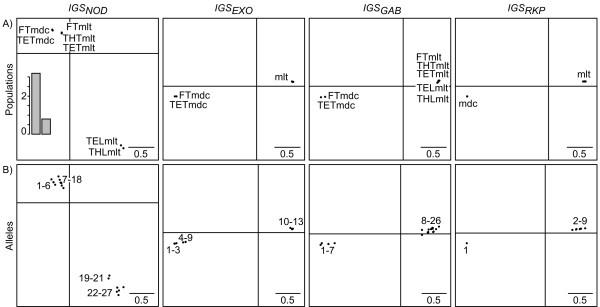
**Application of the DPCoA-MFA to the real data set**. A) Patterns of population differences, and B) allele differences per locus. An eigenvalue barplot is provided at the left-hand corner. Only "mlt" (respectively "mdc") is written when no differentiation can be done on the graphs among *S. meliloti *(respectively *S. medicae*) populations.

It is noteworthy that based on DPCoA-MCoA, the secondary structure is due to a host-plant effect (e.g. *IGS*_*GAB*_) and/or a geographical origin effect (e.g. *IGS*_*EXO*_) discriminating between French and Tunisian populations of *S. meliloti*. Interestingly, the effect of geographical distance on the population structure of *S. meliloti *is not detected by compromise analyses. Because both STATIS and MFA aim at pointing out similarities among loci, these approaches failed at highlighting the secondary structure observed using DPCoA-MCoA (Figure 7B and Figure 8).

There is a clear relationship between the patterns of population differences and the distribution of allelic diversity (Figure 6B). For instance, the two bacterial species did not share any alleles in common, even for the *IGS*_*NOD *_locus. Furthermore, the populations associated with *M. laciniata *did not share any alleles with the populations associated with *M. truncatula *for the *IGS*_*NOD *_locus, resulting in three independent allelic pools belonging respectively to *S. medicae *and the two biovars of *S. meliloti*. Furthermore, the distance between the *IGS*_*NOD *_alleles associated with *M. laciniata *and those associated with *M. truncatula *is very high, almost as high as the distance which separates *S. meliloti *and *S. medicae *on *IGS*_*EXO*_. The particular polymorphism pattern observed for *IGS*_*NOD *_might be explained by both the host-plant selective pressure that acts on *nod *genes and the events of horizontal transfer that affect the *nod *gene cluster [34].

#### Relative effects of distances and frequencies

**Figure 9:**
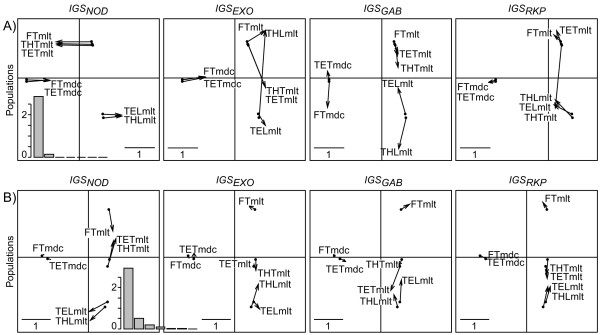
**Effects of allele frequencies and distances in thereal data set**. We applied the DPCoA-MCoA to A) the data set with allele distances without allele frequencies; B) the data set with allele frequencies, without allele distances. In each of the two cases A) and B), each plot gives a comparison between the patterns of the differences among populations given by the compromise over all loci (black dots, start of the arrows) and the individual analyses (end of the arrows).

In order to estimate the relative impacts of allele frequencies and distances in the above results, we applied the DPCoA-MCoA taking into account either sequence divergences without allele frequencies or allele frequencies without sequence divergences (Figure 9). When only sequence divergences are kept, like in the complete analysis, *IGS*_*EXO*_, *IGS*_*GAB*_, and *IGS*_*RKP *_are significantly correlated sharing a strong separation between the species *S. medicae *and *S. meliloti *(correlations vary from 0.81 and 0.93 according to Mantel and are superior to 0.999 according to *Rν*; significance of correlation tests was assessed according to a 0.05 threshold). Regarding the DPCoA-MCoA factorial maps, the population structure is maintained on axis 1, which in that case exhibits 96% of the inter-population diversity. *IGS*_*NOD *_stands out by presenting very distinct alleles according to the host plant. On the second axis, with 4% of the inter-population diversity, the differences between populations according to host plants are maintained for *IGS*_*GAB *_as a secondary structure. Yet, the secondary structures of both *IGS*_*RKP *_and *IGS*_*EXO *_become hardly interpretable. When only the allele frequencies are kept, due to the high differentiation between the two species *S. medicae *and *S. meliloti *for all the loci when allele distances are removed, all the pairwise correlations between loci are significant according to the Mantel statistic (correlations greater than 0.83), and all except *IGS*_*EXO*_-*IGS*_*NOD *_(0.61) and *IGS*_*RKP*_-*IGS*_*NOD *_(0.63) correlations according to the *Rν *statistic. Regarding the DPCoA-MCoA factorial maps, the first axis of all the loci represents the inter-species separation. The difference among populations according to their host plant measured on *IGS*_*NOD *_is relegated to axis 2 representing 12% of the inter-population analysis. Along this axis, all the three other loci *IGS*_*EXO*_, *IGS*_*GAB*_, and *IGS*_*RKP*_ distinguish the French population from the Tunisian populations.

The conclusions which can be drawn from these analyses of the effects of distances and frequencies on the inter-population diversity are as follows. In all of the analyses, the most peculiar locus remains *IGS*_*NOD*_. The high separation of populations according to their host plant is due to distinct and distant alleles for *IGS*_*NOD *_and allele distances for *IGS*_*GAB*_. The differences among *IGS*_*GAB*_, *IGS*_*RKP*_, and *IGS*_*EXO *_are due to differentiation patterns among *S. meliloti *populations. Finally, the distinction between the French and the Tunisian populations mostly relies on allele frequency data.

## Discussion

The MDPCoA approach provides a useful tool for: (i) identifying atypical loci by both tests and factorial maps; (ii) describing differences in population structures between groups of congruent loci by factorial maps; (iii) including evolutionary distances among alleles, which is seldom done.

### Missing data

In all the analyses we performed, the weight of a population is the number of individuals sampled from this population divided by the total number of individuals sampled. Given that we consider several loci, this definition of the weights supposes that we have identified the allelic composition of each individual for all loci. In case of missing allelic data, *i.e*. if the allelic content of some individuals is missing for one or several loci, one should define different weight systems depending on the loci. According to the *g*^th ^locus, the weight of population *i *is the number of characterized individuals from population *i *divided by the total number of characterized individuals. This would lead to *G *different systems of weights, *i.e*. one per locus. Unfortunately, neither STATIS nor the MCoA nor the MFA can support different population weights. Consequently, one will have to assume a similar set of population weights over loci although some data are missing. To overcome this problem, it may be assumed that the weight of a population is the number of individuals sampled from this population divided by the total number of individuals sampled, whether or not the allelic information for all the loci and for all the individuals is available.

Another case of usual missing data is the lack of nucleotide divergence among alleles. In that case, we suggest fixing the distance among any two different alleles equal to 1, so that the DPCoA is equal to the non-symmetric correspondence analysis [11,45]. Furthermore, the inertia of the allelic points per population in the DPCoA "common space" is then equal to the gene diversity index H, introduced by Nei [28], and the inertia of the population points is equal to the gene diversity among populations defined by Nei [28] in its decomposition of gene diversity. The inertia among population points in the best compromise plot and DPCoA-STATIS is a measure of gene diversity among populations averaged over the *G *loci, where the weights given to the loci are not simply uniform but set optimal for synthesizing what is common to the loci. This process gives less weight to outliers and reflects the distances among populations as they are seen by the majority of the loci.

### Effects of frequencies and distances

The effect of frequencies and distances comprises two components: the effect due to sampling error and the effect due to population structure. The effects of sampling error on the component of nucleotide diversity within and between populations have been studied elsewhere [23,46], and might be the object of further research in the context of the mDPCoA.

The relative effects of frequencies and distances on the analysis of population structure depend on the degree of differentiation among the populations under study. In case of low differentiation, population structure is usually due to variations in allelic frequencies. For instance, differences among French and Tunisian populations of *S. meliloti *that are highlighted by *IGS*_*EXO*_, *IGS*_*GAB *_and *IGS*_*RKP *_are due to allelic frequencies. Conversely, as the number of alleles shared by the different population decreases, taking into account the information provided by sequence divergence is crucial to efficiently describe their relationships. For instance, the specific inter-population structure of *IGS*_*NOD *_is mainly due to sequence divergence.

### Pertinence of the correlation tests

Both correlation tests (Mantel and *Rν*) can be non-significant for two reasons: either because of an absence of population structure or because the two loci compared reveal different population structures. As highlighted in a previous section, the estimated *ϕ*_*ST *_parameter and the factorial maps obtained by one of the three versions of the mDPCoA (with MCoA, STATIS or the MFA), can be used to choose among the two alternatives. Concerning the relative interest of the two tests, the *Rν *test is revealed to be more powerful when applied to our simulated data set, so we advocate its use.

### Relative advantages and disadvantages of the three proposed analyses – choice of a method

The three methods are alike in their procedure because they are all based on a compromise. However, they differ in the way the compromise is obtained. With the MCoA, the compromise is built during the definition of the factorial axes. It maximizes the average correlation among the individual analyses and the compromise. With STATIS, the compromise is obtained before going to the core of the multivariate ordination analysis. Here, the compromise maximizes the correlations among the patterns of inter-population diversity provided by the loci. With the MFA, the pieces of information given by the loci are simply added to each other by creating a large table juxtaposing the information on the loci. This last method is the simplest, where pieces of information are simply added. On the other hand, MCoA and STATIS first compare the patterns of inter-population diversity provided by the loci, either for visualizing in a single space the differences among loci or for erasing these differences, and find a best compromise over the loci, respectively.

Unfortunately, the representation of the differences among loci with STATIS is not optimal [15] because STATIS focuses on similarities instead of dissimilarities among loci. Consequently, in comparison to alternative methods, it theoretically lacks an optimal explicability, and an efficient description of the differences in population patterns among loci. The description of the differences among population patterns is thus more precise using MCoA and MFA. Conversely, the main advantage of STATIS over other methods is that it provides a simpler compromise pattern.

The choice among the three methods therefore depends on the goal of the underlying study. If the objective is to obtain the best compromise over the loci, then we advocate the use of DPCOA with STATIS. However, if the objective is to obtain a detailed comparison among the population patterns provided by the *G *loci, then we encourage the use of the DPCoA with the MCoA.

### Complementarity between mDPCoA and other analyses

The mDPCoA could be associated with other tools to study population structure, including the AMOVA, which forms the basis of the DPCoA, Linkage Disequilibrium (LD) statistics, and also recent approaches such as STRUCTURE or CLONAL FRAME.

The AMOVA averages molecular variability over loci to test the existence of differences between populations or groups of populations in terms of both allele frequencies and nucleotide distances among alleles. The Mantel and Rv statistics associated with the mDPCoA use the same information to test the differences between the inter-population structures inferred by several loci.

Both linkage disequilibrium (LD) measures and the mDPCoA aim at assessing whether there is a significant association among the polymorphism patterns observed for different molecular markers. However, LD approaches and mDPCoA differ in several ways. Without discrepancies among the population structures, mDPCoA would fail to detect that different loci evolve independently, even if these are in linkage equilibrium at the population scale. Conversely, in the *Sinorhizobium *spp. data set, the mDPCoA detected that *IGS*_*NOD *_pattern of population differences was drastically different from the ones obtained with *IGS*_*RKP*_, *IGS*_*GAB *_and *IGS*_*EXO*_, suggesting a horizontal gene transfer of *nod *genes between *S. meliloti *bv. meliloti and *S. medicae*. Because of the differentiation between *S. meliloti *and *S. medicae*, LD measures would have failed to detect such a transfer event. Linkage disequilibrium measures and mDPCoA therefore appear as complementary tools to study the influence of sex during the evolution of bacterial lineages.

The mDPCoA is above all a descriptive method, as it does not rely on any assumptions about models of evolution such as linkage equilibrium or selective neutrality. Nevertheless, this analysis pipeline can raise questions that will be investigated using complementary analyses. Thus, demonstrating differences among population structures obtained from different loci raised questions regarding the definition of population boundaries, or the genealogy of both genes and individuals. A consensus population structure could be inferred without any *a priori *knowledge using STRUCTURE, and its efficiency can be confirmed and illustrated using the correlation tests and the graphical outputs of the mDPCoA. CLONAL FRAME is an explanatory method, estimating clonal relationships and looking for key recombination events with a view of finding the mechanisms implied in microevolution [47]. It can be used to gain insights into the history of an atypical locus. Finally, the detection of selection traces and mechanistic experiments can be of great interest to explain mDPCoA results. These different approaches thus complement the mDPCoA, and conversely, the mDPCoA complements these approaches. For instance, both STRUCTURE and CLONAL FRAME imply working on MLS analyses, and the choice of the finite set of loci used in these analyses may be crucial. Each method can be improved by looking at the results returned by the two others. A joint interpretation of the results of the alternative methods may thus allow a better interpretation of the results and lead to a deeper analysis of particular loci for a better understanding of the data.

## Conclusion

All three methods proposed can be used for a better description of inter-population genetic diversity measured over more than one locus. They imply a new reflection on the role of means in measures of diversity: can we work on average information over loci, or do we first need to examine the differences among the patterns of diversity given by the loci? Sometimes, the differences among loci are so high that the compromise obtained by the multivariate analyses will be unstable and the use of averaged information can hamper interpretation. This issue is related to the question raised decades ago: can we build a unique, very synthetic measure of biodiversity, or do we have to make up our mind to define several conflicting measures? As it is based on multivariate analyses, the multiple DPCoA in its three forms can be used to analyze large data sets. It allows a comparison of genetic diversity measured on various loci. It complements existing tools such as AMOVA and linkage disequilibrium measures. It is used here on molecular data because it is in genetics the question of congruence among markers was raised several years ago. We illustrated this procedure using a limited but complex sequence database. The method will have to be tested on other data sets, yet the results are already very promising. Moreover, mDPCoA is potentially more general than we presented here since it can be extended to any data set where pairs of matrices comprise a matrix with abundance or presence/absence and a matrix of dissimilarities. Further applications in ecology could thus be considered, such as the description of inter-community diversity based on both genotypic and phenotypic features.

## Abbreviations

AMOVA, Analysis of MOlecular Variance; bv., biovar; DPCoA, Double Principal Coordinate Analysis; FTmdc, Population sampled at Sainte Colombe l'Eglise in France from *M. truncatula *nodules which include *S. medicae *isolates; FTmlt, Population sampled at Sainte Colombe l'Eglise in France from *M. truncatula *nodules which include *S. meliloti *bv. meliloti isolates; IGS, Intergenic spacers; LD, Linkage disequilibrium; MCoA, Multiple Co-inertia Analysis; mDPCoA, multiple Double Principal Coordinate Analysis; MFA, Multiple Factorial Analysis; MLS, Multilocus Sequencing; PCA, Principal Component Analysis; STATIS, comes from a French expression "structuration des tabeaux à trois indices de la statistique" which means: structuration of the tables characterized by three statistical modes; TELmlt, Population sampled in Tunisia at Enfidha from *M. laciniata *nodules which include *S. meliloti *bv. medicaginis isolates; TETmdc, Population sampled in Tunisia at Enfidha from *M. truncatula *nodules which include *S. medicae *isolates; TETmlt, Population sampled in Tunisia at Enfidha from *M. truncatula *nodules which include *S. meliloti *bv. meliloti isolates; THLmlt, Population sampled in Tunisia at Hadjeb from *M. laciniata *nodules which include *S. meliloti *bv. medicaginis isolates; THTmlt, Population sampled in Tunisia at Hadjeb from *M. truncatula *nodules which include *S. meliloti *bv. meliloti isolates.

## Authors' contributions

SP developed the methodology and applied it to the data. XB performed the simulations and characterized *Sinorhizobium *populations. He interpreted the results. Both authors contributed equally to the discussion. Both authors read and approved the final draft.

## Supplementary Material

Additional file 1 Functions in R to perform multiple DPCoA. The file is called "mdpcoa.R". It can be read by the R software which can be downloaded free of charge, and one can refer to the Additional file 2 for explanation on how to use it. Click here for file

Additional file 2 Instructions for performing multiple DPCoA in R. The file is called "Instruction.pdf". It describes in step by step detail how to use R to perform a multiple DPCoA using the real data set in this paper. Click here for file

Additional file 3 Description of the real data set. The complete sampling procedure is given together with a description of within-population diversity. Click here for file

Additional file 4 DNA sequences for IGSNOD. Sequences are in "FASTA" format. The File is named "NOD.aa". See Additional file 2 for explanation on how to use this file. Click here for file

Additional file 5 DNA sequences for IGSEXO. Sequences are in "FASTA" format. The File is named "EXO.aa". See Additional file 2 for explanation on how to use this file. Click here for file

Additional file 6 DNA sequences for IGSGAB. Sequences are in "FASTA" format. The File is named "GAB.aa". See Additional file 2 for explanation on how to use this file. Click here for file

Additional file 7 DNA sequences for IGSRKP. Sequences are in "FASTA" format. The File is named "RKP.aa". See Additional file 2 for explanation on how to use this file. Click here for file
