# Supplementary material for: New analysis for consistency among markers in the study of genetic diversity: development and application to the description of bacterial diversity
Source: BMC Evol Biol. 2007 Sep 3;7:156. doi: 10.1186/1471-2148-7-156 (PMC2082430; doi:10.1186/1471-2148-7-156)
Supplement: Additional file 2 — Instructions for performing multiple DPCoA in R. The file is called "Instruction.pdf". It describes in step by step detail how to use R to perform a multiple DPCoA using the real data set in this paper. [file 1471-2148-7-156-S2.pdf]

Additional file 2 - Instructions for performing multiple DPCoA in R

R is a free software available at http://lib.stat.cmu.edu/R/CRAN

It includes hundreds of packages in many scientific areas.

Create a working directory, and be carrefull to define the right working directory in your R console (see in the tool bar, → file → choosing directory).

For analyzing your genetic data and performing the multiple DPCoA, you need to load two packages from R: 'ape' and 'ade4'. Write the following instructions on your R console:

```
library(ade4)
library(ape)
```

In your working directory, copy the file 'mdpcoa.R' in your working directory (Additional file 1) and load it:

```
source("mdpcoa.R")
```

This file contains three functions: one for the preparation of the data (`prep.mdpcoa`), the second for the numerical calculations (`mdpcoa`), and the third for graphical displays (`kplotX.mdpcoa`). These three functions are described below.

1/ IF YOUR DATA SET CONTAINS LISTS OF DNA SEQUENCES

Create a folder (for our case study, name it "dna" using small characters) in your working directory, open it and copy your DNA files.

With our case study, copy the files 'NOD.aa', 'EXO.aa', 'GAB.aa', and 'RKP.aa' (Additional files 4 to 7) in the "dna" folder. Each file corresponds to a locus. It contains the sequences in the format 'FASTA'. Note that the library ape can handle various format of DNA file (see the manual of this library for more information). Next we need to load the four files. To obtain a factor giving the name of the population to which each sequence belongs load the file "pop.txt":

```
pop <- as.factor(read.table("pop.txt")[,1])
```

Use the function "prep.mdpcoa" to prepare the data: If your data set contains lists of DNA sequences, the function will provide you with the list of matrices giving the abundance of each allele in populations, the list of distances among alleles, and the list of the allele DNA sequences.

The function prep.mdpcoa has five parameters:

```
prep.mdpcoa(folder, pop, format, model, ...)

folder is a character string giving the path through the folder which
     contains the DNA sequences. This folder should contain only the
     sequences of interest for the study.

pop is a factor giving the name of the population to which each sequence
     belongs.
```

```
format is a character string specifying the format of the DNA sequences.
     Three choices are possible: '"interleaved"', '"sequential"', or
     '"fasta"', or any unambiguous abbreviation of these.

model is a vector giving the model to be applied for the calculations of
     the distances for each locus. One model should be attributed to each
     locus, given that the loci are in alphabetical order. The models can
     take the following values: "raw", "JC69", "K80" (the default), "F81",
     "K81", "F84", "BH87", "T92", "TN93", "GG95", "logdet", or "paralin".
     See the help documentation for the function "dist.dna" of ape for a
     describtion of the models.

... designs further arguments passed to the read.dna function
```

We apply now this function to our real data set:

```
dat <- prep.mdpcoa("dna", pop, model = c("F84", "F84", "F84", "F81"),
     pairwise.deletion = TRUE)
```

The object “dat” is a list of three sublists:

- `dat$sam`: is a list of data frames with the populations as columns, alleles as rows and abundances as entries. Each table corresponds to a locus.
- `dat$dis`: is a list of objects of class ‘dist’, corresponding to the distances among alleles
- `dat$alleleseq`: is a list of objects of class ‘dna’ providing the DNA sequence of each allele for all the loci.

We will now stock the abundance matrices and the distances in the following two objects, and proceed to the mDPCoA.

```
sam <- dat$sam
dis <- dat$dis
```

The distances should be Euclidean. Several transformations exist to render a distance object Euclidean (see functions `cailliez`, `lingoes` and `quasieuclid` in the ade4 package). Here we use the quasieuclid function.

```
dis <- lapply(dis, quasieuclid)
```

2/ A UNIQUE FUNCTION WITH A LARGE SET OF OPTIONS:

As indicated above, the functions developed for the multiple DPCoA are available in the Additional file 1. If it has not yet been done, copy this file in your working directory and write the following instruction:

```
source("mdpcoa.R")
```

This file contains three functions, the function `prep.mdpcoa` that we have just seen, a function for the numerical calculations (`mdpcoa`), and the third function for graphical displays (`kplotX.mdpcoa`). The two last functions have the following expressions:

```
mdpcoa <- function(msamples, mdistances = NULL, method = c("mcoa", "statis",
          "mfa"), option = c("inertia", "lambda1", "uniform", "internal"),
          scannf = TRUE, nf = 3, full = TRUE, nfsep = NULL, tol = 1e-07)

kplotX.mdpcoa <- function(object, xax = 1, yax = 2, mfrow = NULL,
          which.tab = 1:length(object$nX), includepop = FALSE, clab = 0.7,
          cpoi = 0.7, unique.scale = FALSE, csub = 2, possub = "bottomright")
```

In these two expressions, the parameters are:

`msamples`	A list of data frames with the populations as columns, alleles as rows and abundances as entries. All the tables should have equal numbers of columns (populations). Each table corresponds to a locus.
`mdistances`	A list of objects of class ‘dist’, corresponding to the distances among alleles. The order of the loci should be the same in msamples as in mdistances.
`method`	One of the three possibilities: “mcoa”, “statis”, or “mfa”. If a vector is given, only its first value is considered.
`option`	One of the four possibilities for normalizing the population coordinates over the loci: "inertia", "lambda1", "uniform", or "internal". These options are used with MCoA and MFA only.
`scannf`	a logical value indicating whether the eigenvalues bar plots should be displayed
`nf`	if scannf is FALSE, an integer indicating the number of kept axes for the multiple analysis
`full`	a logical value indicating whether all the axes should be kept in the separated analyses (one analysis, DPCoA, per locus)
`nfsep`	if full is FALSE, a vector indicating the number of kept axes for each of the separated analyses
`tol`	a tolerance threshold for null eigenvalues (a value less than tol times the first one is considered as null)
`object`	an object of class ‘mdpcoa’
`xax`	the number of the x-axis
`yax`	the number of the y-axis
`mfrow`	a vector of the form 'c(nr,nc)', otherwise computed by as special own function 'n2mfrow'
`which.tab`	a numeric vector containing the numbers of the loci to analyse
`includepop`	a logical indicating if the populations must be displayed. In that case, the alleles are displayed by points and the populations by labels
`clab`	a character size for the labels
`cpoi`	a character size for plotting the points, used with 'par("cex")'*cpoint. If zero, no points are drawn
`unique.scale`	if TRUE, all the arrays of figures have the same scale
`csub`	a character size for the labels of the arrays of figures used with 'par("cex")*csub'
`possub`	a string of characters indicating the sub-title position ("topleft", "topright", "bottomleft", "bottomright")

An object obtained by the function `mdpcoa` has two classes. The first one is “`mdpcoa`” and the second is either “`mcoa`”, or “`statis`”, or “`mfa`”, depending on the method chosen. Consequently, other functions already available in ade4 for displaying graphical results can be used:

With MCoA,

- `plot.mcoa`: this function displays (1) the differences among the populations according to each locus and the compromise, (2) the projection of the principal axes of the individual analyses onto the synthetic variables (noted **V** in the main text of the paper), (3) the projection of the principal axes of the individual analyses onto the co-inertia axes (noted $\mathbf{U}_g$ in the main text of the paper), (4) the squared vectorial covariance among the coinertia scores ($\mathbf{L}_{Y_g} = \sqrt{\pi_g}\mathbf{Y}_g\mathbf{U}_g$) and the synthetic variables (**V**);

- `kplot.mcoa` : this function divides previous displays (figures 1, 2, or 3 described in `plot.mcoa`) by giving one plot per locus.

With STATIS,

- `plot.statis`: this function displays (1) the scores of each locus according to the two first eigenvectors of the matrix *Rv*, (2) the scatter diagram of the differences among populations according to the compromise, (3) the weight (α_g) attributed to each locus in abscissa and the vectorial covariance among each individual analysis ($\mathbf{E}_g$ with the notations in the main text of the paper) and the compromise analysis ($\mathbf{E}/\|\mathbf{E}\|$) in ordinates, (4) the covariance between the principal component inertia axes of each locus and the axes of the compromise space ($B_r^{-1/2}\mathbf{U}_g$);
- `kplot.statis`: this function displays for each locus the projection of the principal axes onto the compromise space.

With MFA,

- `plot.mfa`: this function displays (1) the differences among the populations according to each locus and the compromise, (2) the projection of the principal axes of the individual analyses onto the compromise, (3) the covariance between the principal component inertia axes of each locus and the axes of the compromise space, (4) for each axis of the compromise, the amount of inertia conserved by the projection of the individual analyses onto the common space.
- `kplot.mfa`: this function displays for each locus the projection of the principal axes and populations onto the compromise space.

Further comments on these functions will be found in the help files of ade4.

3/ APPLICATION

DPCOA and MCOA :

The calculations are saved in an object called “mdpcoa1”:

```
mdpcoa1 <- mdpcoa(sam, dis, "mcoa", scannf = FALSE, full = FALSE,
    nfsep = rep(2,4))
```

The following plots correspond to Figure 6 in the article. It shows the discrepencies among loci in the population patterns they provide. The first plot is the compromise. In the next four plots, arrows connect the positions of the populations according to the compromise with their positions according to the defined locus.

```
kplot.mcoa(mdpcoa1)
```

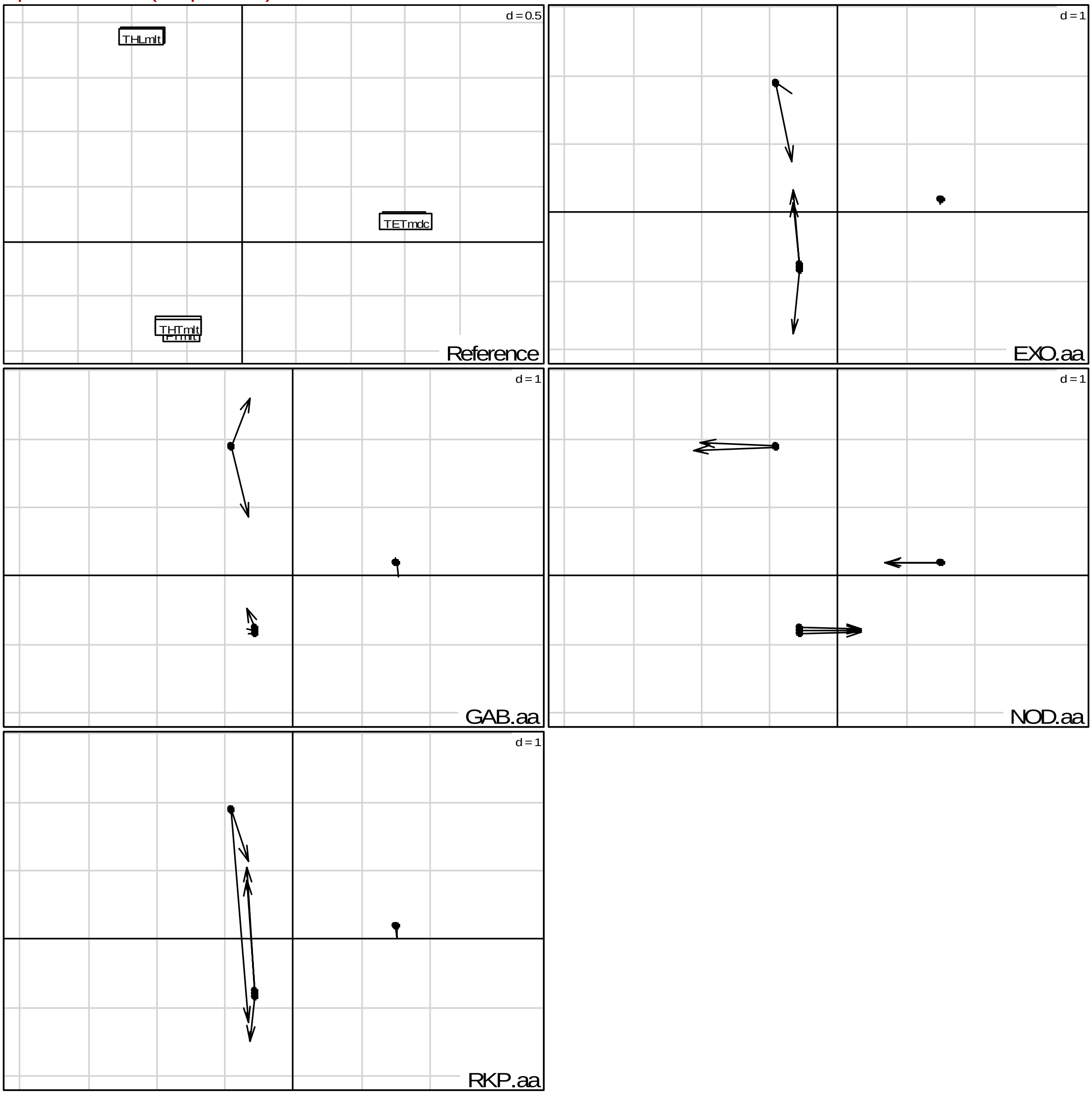

The following plot corresponds to Figure 6b in the article. It shows the position of the alleles on the common space.

```
kplotX.mdpcoa(mdpcoa1)
```

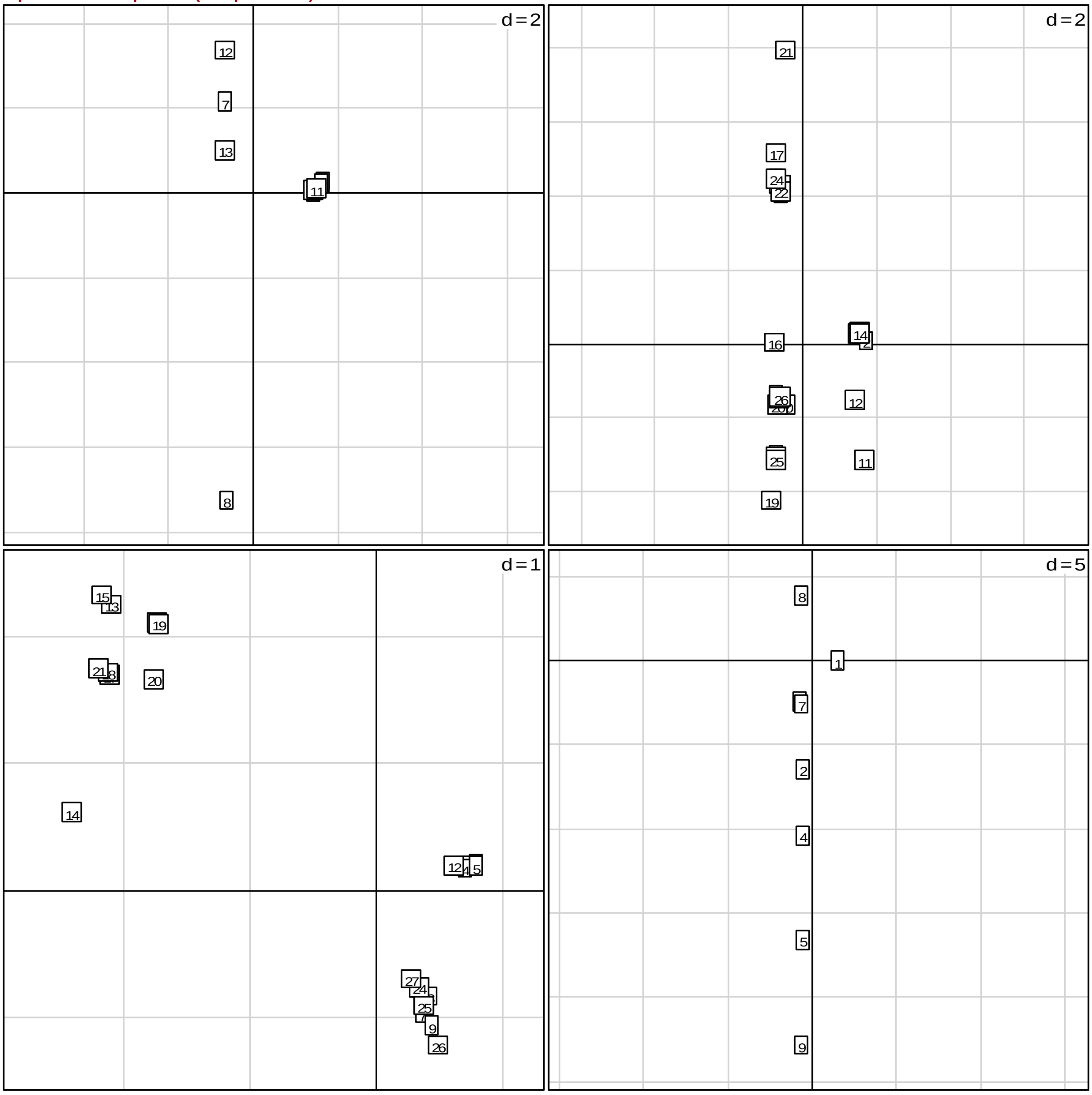

Interestingly, one can combine the two previous figures to superimpose the populations and their alleles:

```
kplotX.mdpcoa(mdpcoa1, include = TRUE)
```

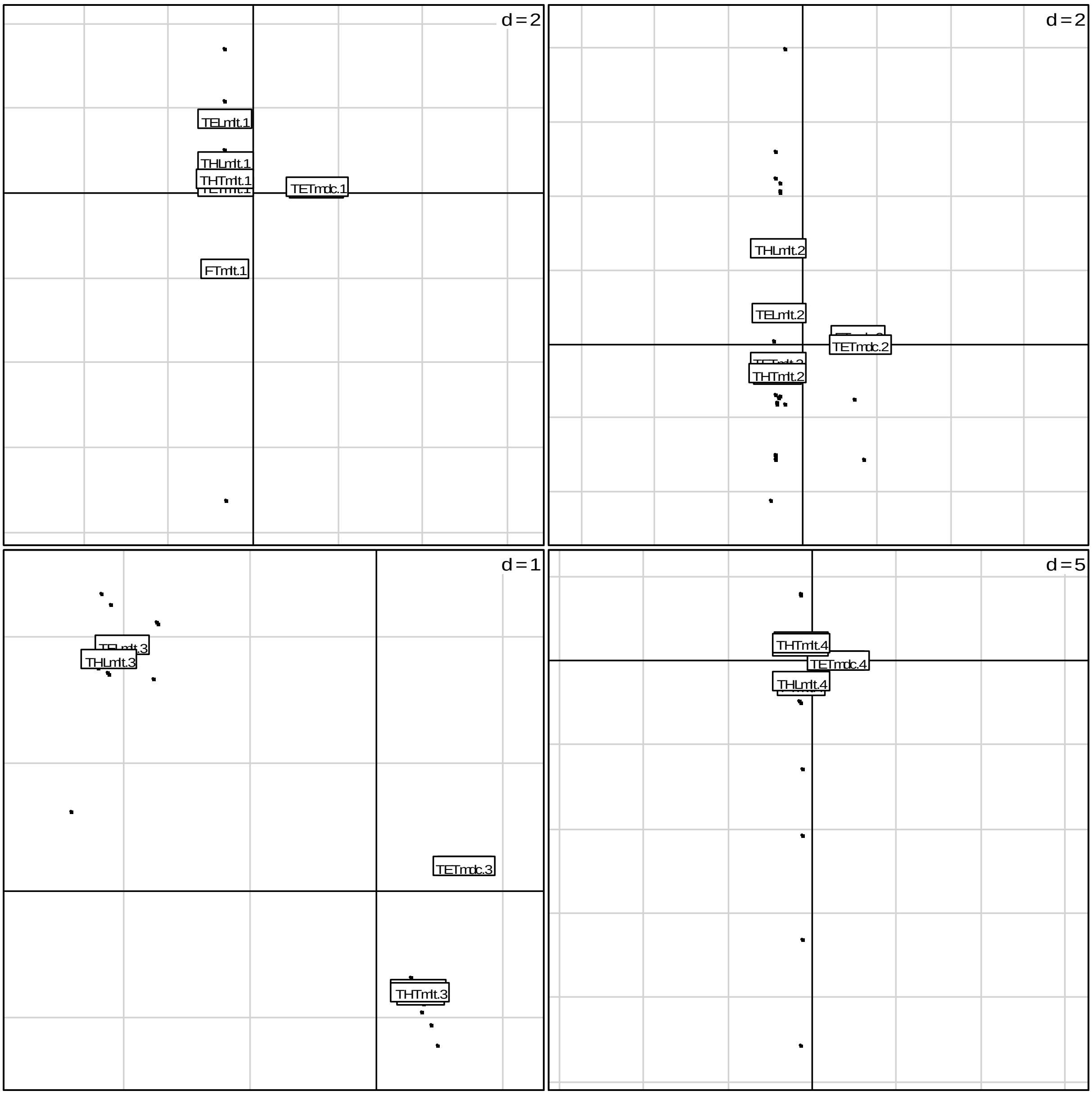
d = 2
TELmlt.1
THLmlt.1
THTmlt.1
TETmdc.1
FTmlt.1
d = 2
THLmlt.2
TELmlt.2
TETmdc.2
THTmlt.2
d = 1
THLmlt.3
TETmdc.3
THTmlt.3
d = 5
THTmlt.4
TETmdc.4
THLmlt.4

DPCOA and STATIS:

The calculations are now saved in an object called “mdpcoa2”:

```
mdpcoa2 <- mdpcoa(sam, dis, "statis", scannf = F, full = FALSE,
    nfsep = rep(2,4))
```

In the following plot, one can recognize in the top displays, the figure 7. It shows on the top left-hand corner the scores of each locus according to the two first eigenvectors of the matrix *Rv* with the eigenvalue barplot of *Rv*, and on the top right-hand corner the scatter diagram of the differences among populations according to the compromise with the eigenvalue barplot of the whole analysis (variance among population points on the compromise). As said on page 4, the two last plots display (on the bottom left-hand corner) the weight (α_g) attributed to each locus in abscissa and the vectorial covariance among each individual analysis ($\mathbf{E}_g$ with the notations in the main text of the paper) and the compromise analysis ($\mathbf{E}/\|\mathbf{E}\|$) in ordinates, (on the bottom right-hand corner) the covariance between the first principal component inertia axis of each locus and the two first axes of the compromise space ($B_r^{-1/2}\mathbf{U}_g$), with the eigenvalue barplot of the whole analysis.

```
plot.statis(mdpcoa2)
```

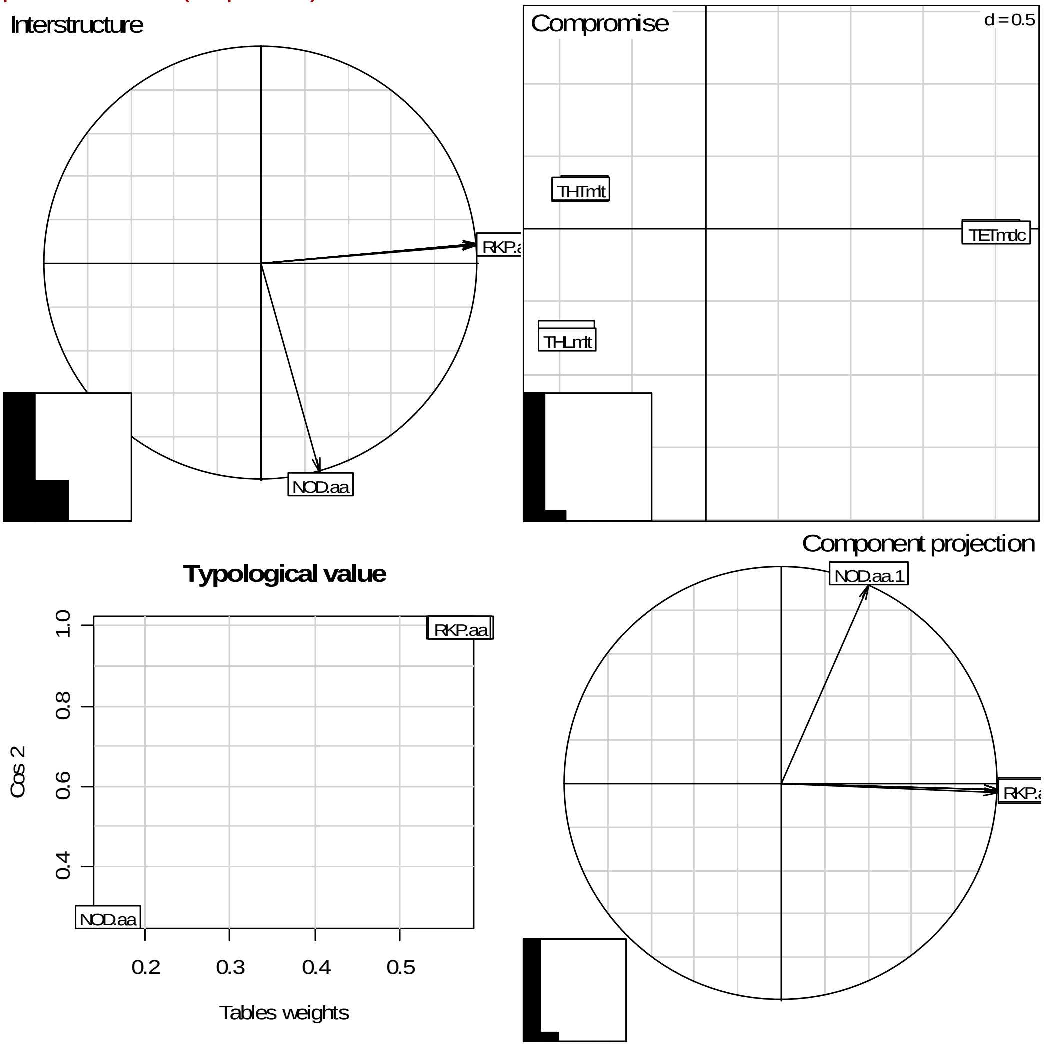

The following figure provides, for each locus, a superimposition of the populations (labels) and their alleles (points) in the compromise space.

```
kplotX.mdpcoa(mdpcoa2, include = TRUE)
```

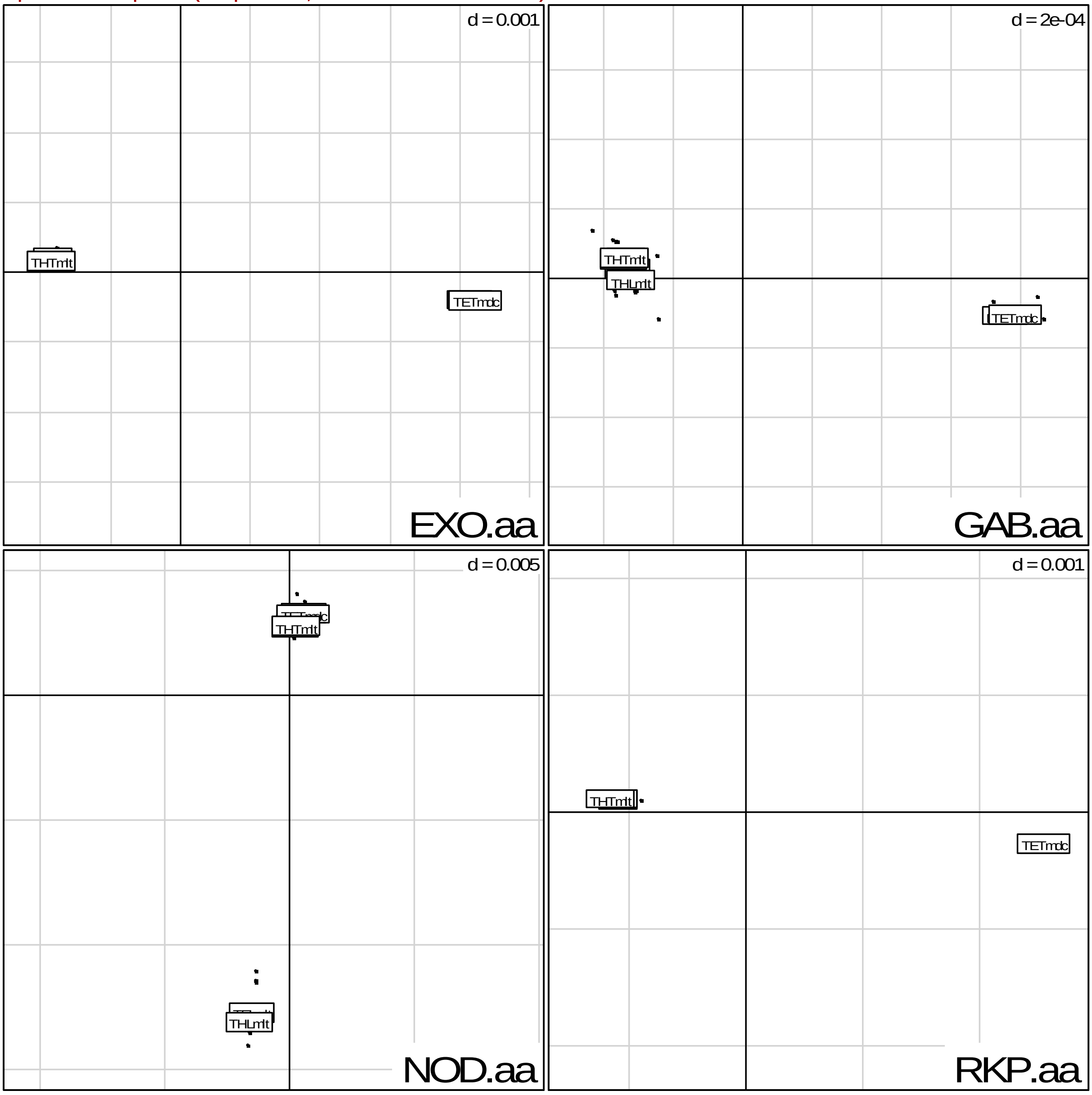

DPCOA and MFA :

For the last analysis, the calculations are saved in an object called “mdpcoa3”:

```
mdpcoa3 <- mdpcoa(sam, dis, "mfa", scannf = F, full = FALSE,
    nfsep = rep(2,4))
```

In the following plot, we have (top right-hand corner) the differences among the populations according to each locus and the compromise, (top left-hand corner) the projection of the principal axes of the individual analyses on the compromise, (bottom left-hand corner) the covariance between the principal component inertia axes of each locus and the axes of the compromise space, (bottom right-hand corner) for each axis of the compromise, the amount of inertia conserved by the projection of the individual analyses on the common space. The three eigenvalue barplots are equal. They measure for each principal axis, the amount of variance among population coordinates according to the compromise.

```
plot.mfa(mdpcoa3)
```

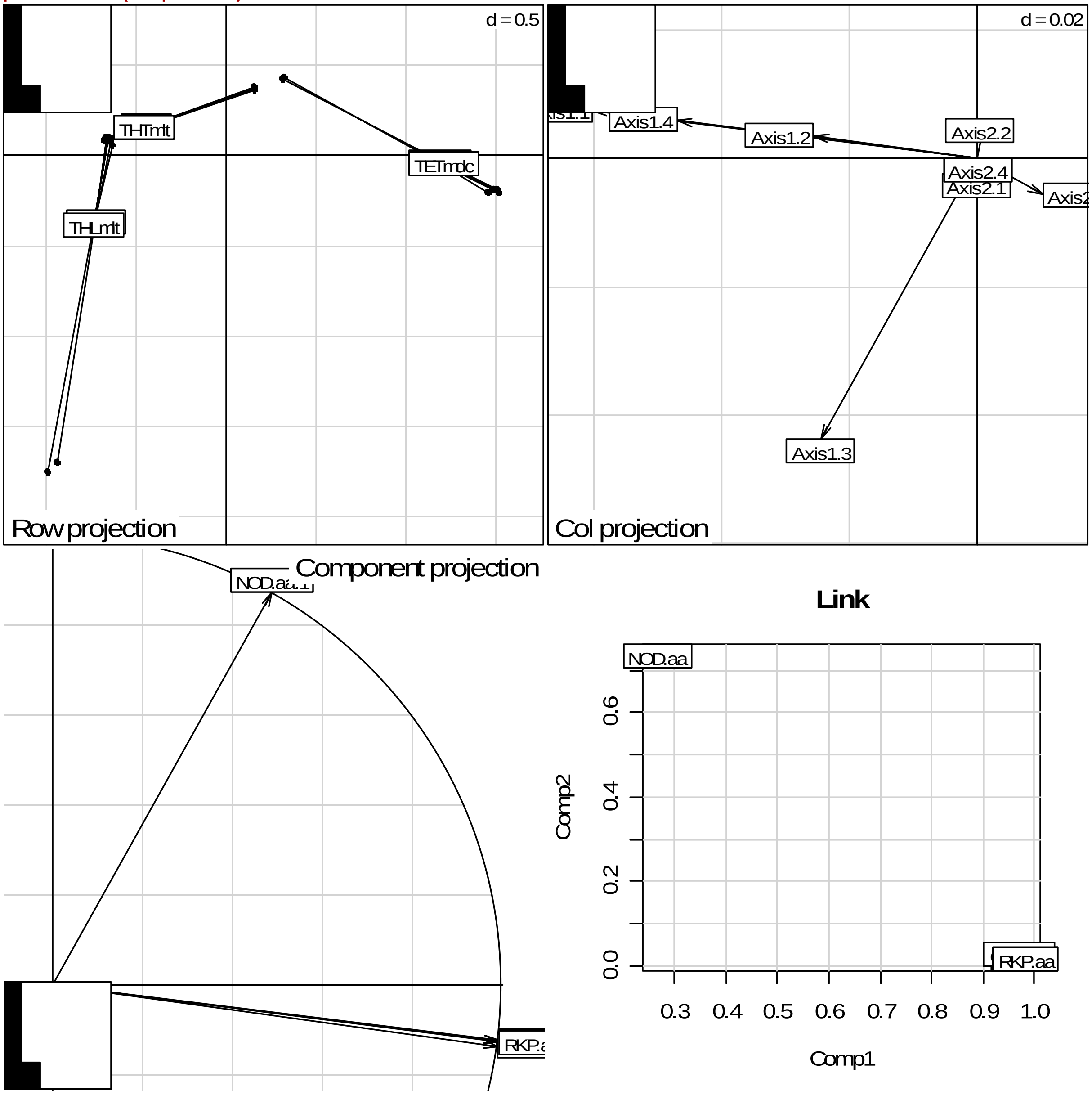

The figure below displays for each locus the projection of the principal axes and populations onto the compromise space.

```
kplot.mfa(mdpcoa3, row.names = TRUE, clab=0.7)
```

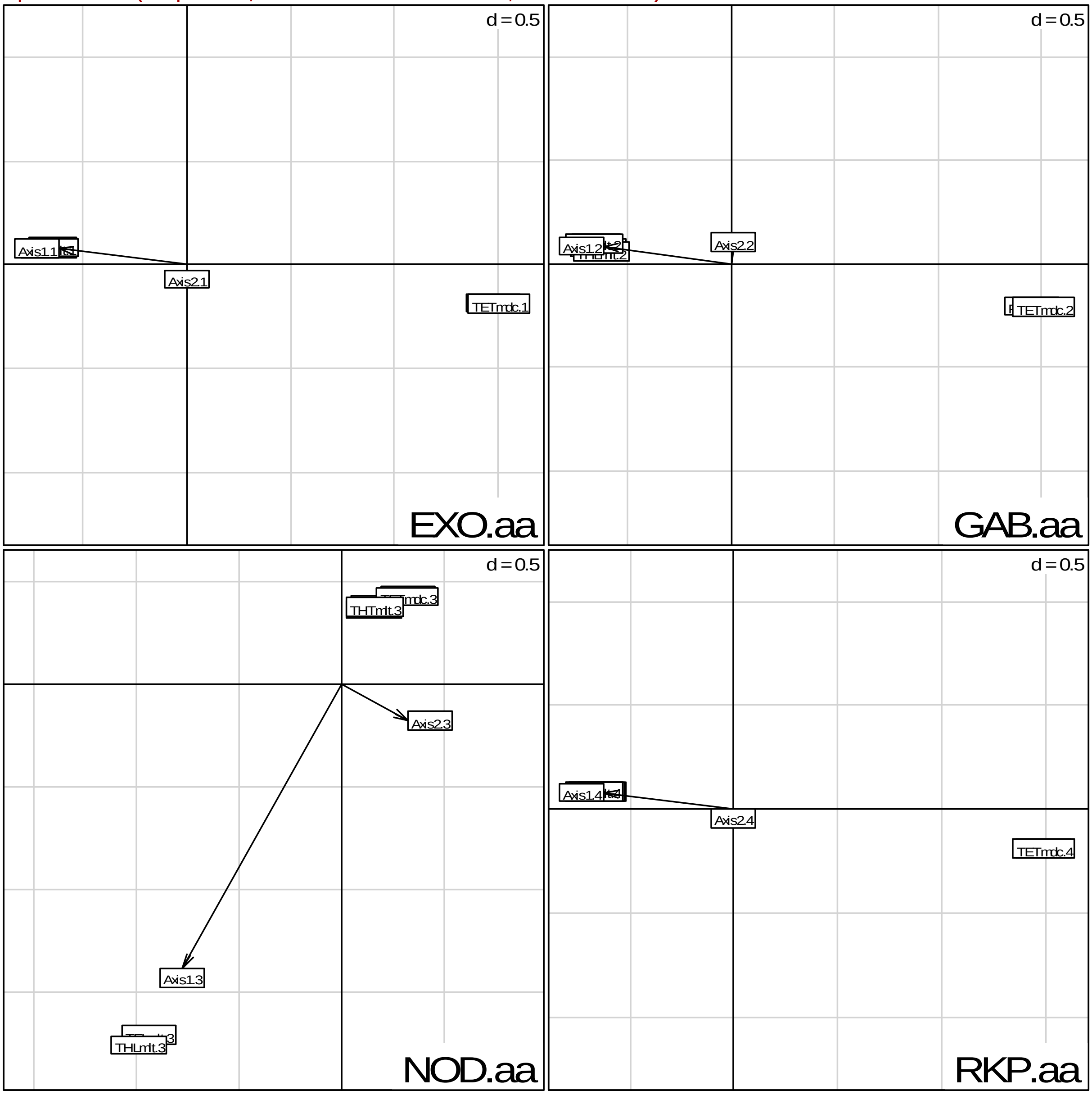

The following plot shows the position of the alleles on the common space.

```
kplotX.mdpcoa(mdpcoa3)
```

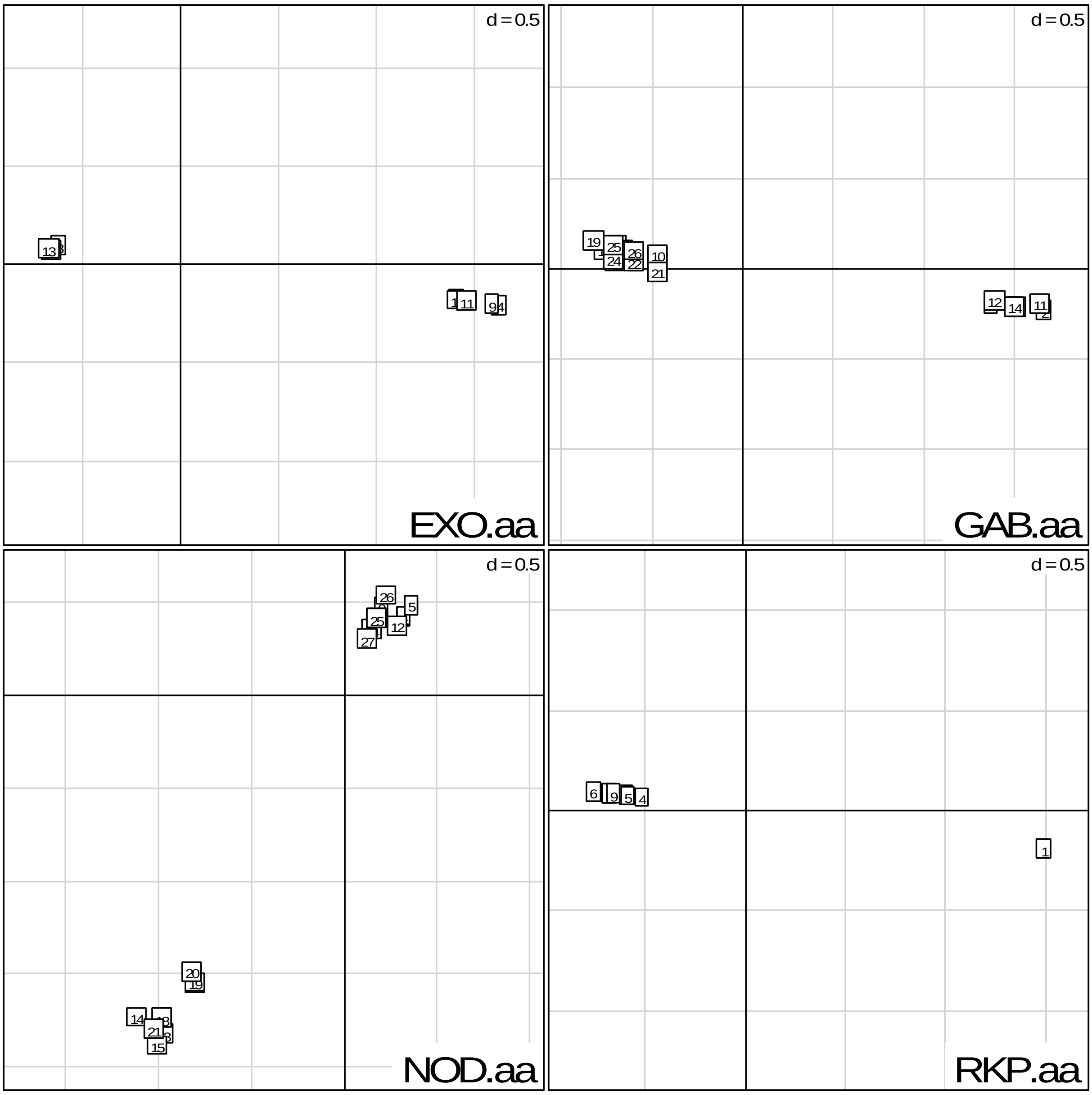

Interestingly, one can combine the two previous figures to superimpose the populations and their alleles:

```
kplotX.mdpcoa(mdpcoa3, include = TRUE)
```

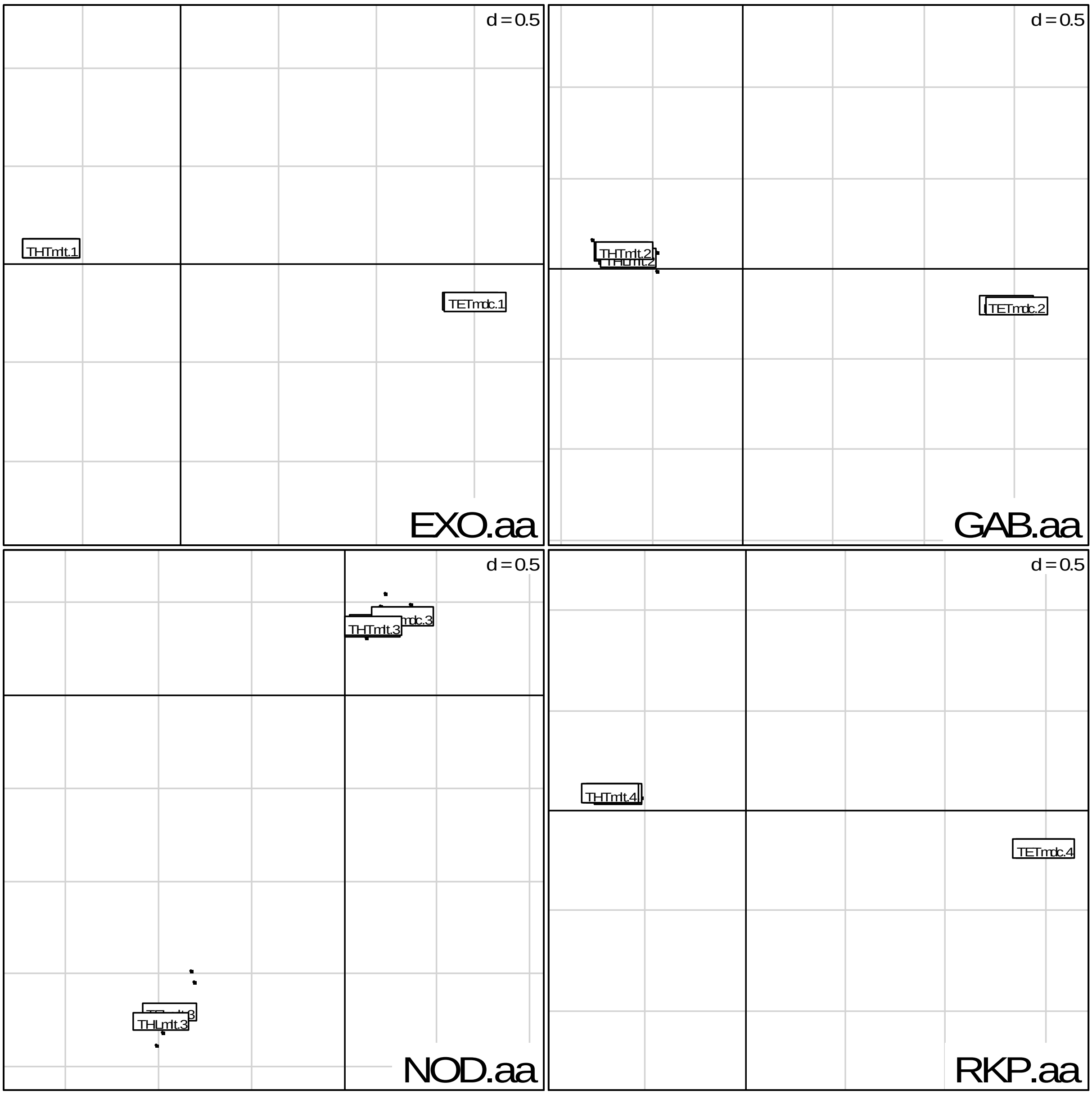
d = 0.5
THTmlt.1
TETmdc.1
EXO.aa
d = 0.5
THTmlt.2
TETmdc.2
GAB.aa
d = 0.5
THTmlt.3
THLmlt.3
NOD.aa
d = 0.5
THTmlt.4
TETmdc.4
RKP.aa
