# Supplementary material for: New analysis for consistency among markers in the study of genetic diversity: development and application to the description of bacterial diversity
Source: BMC Evol Biol. 2007 Sep 3;7:156. doi: 10.1186/1471-2148-7-156 (PMC2082430; doi:10.1186/1471-2148-7-156)
Supplement: Additional file 3 — Description of the real data set. The complete sampling procedure is given together with a description of within-population diversity. [file 1471-2148-7-156-S3.pdf]

Additional file 2 - Description of the complete sampling procedure for the real data set

One liter of top soil was sampled at Sainte Colombe l'Eglise in the south of France (longitude 02°45'26" East – latitude 42°37'59" North). The soil sample was put into contact with individuals of *M. truncatula.* Two one liter samples of top soil were obtained from two localities in Tunisia which are included in a semi-arid bioclimatic area: Enfidha (longitude 10°23' East – latitude 36°07' North) and Hadjeb (longitude 9°33' East – latitude 35°24' North). Two plant species were used to sample rhizobia *ex situ* from each of these Tunisian soils: i) 40 individuals of *M. truncatula* (Jemalong A17 line), ii) 20 individuals of *M. laciniata* (bulk of genotypes). We selected from the whole data set bacterial genotypes which were fully assessed for the four loci.

Isolation and culture of bacteria

The soil sample was mechanically homogenized before the experiment and aliquots were put into contact with seed germinations. Gibson tubes were previously nine-tenth filled with vermiculite and 25 ml of nitrogen-free plant nutrient solution. Tubes were autoclaved, and then completed with a soil aliquot. Seeds were surface sterilized with calcium hypochlorite 5% (weight/volume) for 5 minutes and rinsed with sterile water. Seeds were germinated for 72 hours on 1% (w/v) agar medium. Each plant was transferred individually into a Gibson tube which was placed in a growth chamber at 22°C day / 18°C night, with a 16-hour photoperiod and 50-60% relative humidity. After two months, symbiotic effectiveness was estimated by visual observation of plant vigor, foliage and nodule color, and all nodules from each plant were harvested. One or two nodules per plant were sterilized using hypochlorite 1% (w/v) for three minutes, then rinsed three times in sterile water. The isolation of one bacterial strain per nodule was performed using three successive sub-cultures on YEMA (yeast extract manitol agar) solid medium of an isolated colony starting from crushed nodules.

Bacterial strains were preserved at -80°C in YEM medium supplemented with a half volume of 60% (volume/volume) glycerol. Each bacterial isolate was then grown in 20 ml YEM medium.

DNA extraction and Sequencing

Sequences of PCR/sequencing primers are provided in Table 1.

Table 1. Primers used in this study

Genome location[a]	Primers	Sequence 5'→3'	T_m (°C)	PCR product size (bp)
IGS_{RKP} (*rkpA – rkpU*)	RKP1	AGGCATGCACGCCCTATGA	58.8	599
	RKP2	GCCATCGACATCTACAATATCAA	57.1	
IGS_{NOD} (*nodE – nodG*)	NOD1	CAGTTCTGGCATTCAAGC	53.7	579
	NOD2	CCCCTCCTATGGCTCCTGAT	61.4	
IGS_{GAB} (*gabD5 –Sma1850*)	GAB1	CATGACCAAAGACCGCTTCC	59.4	658
	GAB2	GCATGATCGGCCTCAACAC	58.8	
IGS_{EXO} (*exoP – thiD*)	EXOmeliloti1	CAACAAGACGGATATGAACGAA	56.5	494
	EXOmeliloti2	GTGGTGGAAGGATTGACTGC	59.4	
	EXOmedicae1	CATGAACGAGCTGGGCAAAT	57.3	250
	EXOmedicae2	CTGGTCGAAGCGGCAAAA	56.0	

[a] Genome locations refer to the genome of *S. meliloti* strain 1021

One milliliter of liquid culture was washed twice in an Eppendorf micro-tube by centrifugation (15000 g, 4 min) and the pellet re-suspended in 750 µl of sterile water. One hundred microliters of this solution was incubated for two hours with 20 µl of 1 $mg.ml^{-1}$ proteinase K and 100 µl of tris-HCl (10 mM, pH 8.3). After boiling, this mixture was used as the DNA matrix. DNA amplification was performed using a Perkin-Elmer 2400 thermocycler in 25 µl volume, including 1 µl of DNA matrix, 200 µM of each dNTP, 0.8 µM of each primer, 1.5 mM of $MgCl_2$, 1X buffer supplied by the *Taq* polymerase manufacturer, and 1.25 U of Invitrogen *Taq* polymerase. We used a touch-down program including an initial denaturation stage (96°C, 4 min), 20 cycles of denaturation (96°C, 30 s), annealing (annealing temperature decrease steadily from 60°C to 50°C in 20 cycles, 30 s), elongation (72°C, 1 min)

and 20 cycles of denaturation (96°C, 30 s), annealing (50°C, 30 s) and elongation (72°C, 1 min). PCR products were cut out from a 1% (w/v) agarose electrophoresis gel, purified with a QIAquick Gel extraction kit (Qiagen) and sequenced on one strand. For French samples, sequencing reactions were performed using the ABI Prism BigDye Terminator Cycle sequence Kit (Applied Biosystem) and analyzed on an Applied Biosystem model 310 DNA sequencer. For Tunisian populations sequencing reactions were performed using the DYEnamic ET Terminator Kit (Amersham Bioscience) and analyzed on an Amersham Bioscience model Megabase 1000 DNA sequencer.

Nucleotides sequence accession numbers

All sequences have been deposited in the GenBank data base.

Table 2. Accession numbers

	French samples	Tunisian samples
IGS_{NOD}	DQ405403–DQ405466, DQ405593–DQ405641	DQ405467-DQ405592
IGS_{EXO}	DQ405994–DQ406103	DQ406104- DQ406229
IGS_{GAB}	DQ406342–DQ406392, DQ406519–DQ406567	DQ406393- DQ406518
IGS_{RKP}	DQ405768–DQ405882	DQ405642- DQ405767

Population diversity

For each loci, four parameters of population diversity are given: allele richness, gene diversity, the average distance among alleles, and the observed nucleotide diversity scaled by the maximum value given the set of alleles [1, 2].

Table 3. Population diversity

IGS$_{NOD}$	Richness	Gene diversity	Average distance	Nucleotide diversity
FTmdc	5	0.493	0.00187	0.00027
TETmdc	3	0.565	0.00222	0.00481
FTmlt	6	0.559	0.00679	0.00297
TETmlt	4	0.490	0.00288	0.00051
THTmlt	7	0.571	0.00393	0.00306
TELmlt	8	0.830	0.01830	0.05009
THLmlt	7	0.730	0.01703	0.03742

IGS$_{EXO}$	Richness	Gene diversity	Average distance	Nucleotide diversity
FTmdc	6	0.677	0.00817	0.01106
TETmdc	7	0.795	0.00634	0.01141
FTmlt	2	0.487	0.03575	0.06183
TETmlt	3	0.392	0.02385	0.04153
THTmlt	3	0.346	0.02385	0.03899
TELmlt	3	0.185	0.02675	0.01244
THLmlt	2	0.255	0.03575	0.03239

IGS$_{GAB}$	Richness	Gene diversity	Average distance	Nucleotide diversity
FTmdc	3	0.572	0.00210	0.00746
TETmdc	7	0.765	0.00566	0.03339
FTmlt	7	0.432	0.01033	0.03883
TETmlt	9	0.806	0.01343	0.09763
THTmlt	8	0.749	0.00953	0.06973
TELmlt	5	0.690	0.01098	0.07279
THLmlt	5	0.720	0.01027	0.05087

IGS$_{RKP}$	Richness	Gene diversity	Average distance	Nucleotide diversity
FTmdc	1	0.000	0.00000	0.00000
TETmdc	1	0.000	0.00000	0.00000
FTmlt	3	0.522	0.00447	0.00789
TETmlt	4	0.462	0.00669	0.01711
THTmlt	6	0.475	0.00671	0.01520
TELmlt	3	0.265	0.00670	0.01845
THLmlt	2	0.375	0.01006	0.03085

References

1. Champely S, Chessel D: **Measuring biological diversity using Euclidean metrics.** *Environ Ecol Stat* 2002, **9**:167-177.
2. Pavoine S, Ollier S, Pontier D: **Measuring diversity from dissimilarities with Rao's quadratic entropy: are any dissimilarity indices suitable?** *Theor Popul Biol* 2005, **67**(4):231-239.
